# RNautophagic regulation of DNMT3a‐dependent DNA methylation by Linc00942 enhances chemoresistance in gastric cancer

**DOI:** 10.1002/ctm2.1337

**Published:** 2023-07-21

**Authors:** Liyuan Zhu, Yiran Zhu, Fang Li, Yuan Meng, Hanying Wang, Wenxia Xu, Jingfeng Luo, Xian Wang, Lifeng Feng, Hongchuan Jin

**Affiliations:** ^1^ Laboratory of Cancer Biology, Key Lab of Biotherapy in Zhejiang Province, Cancer Center of Zhejiang University, Sir Run Run Shaw Hospital, School of Medicine Zhejiang University Hangzhou China; ^2^ Department of Medical Oncology Sir Run Run Shaw Hospital School of Medicine Zhejiang University Hangzhou China

**Keywords:** chemoresistance, DNMT3a, Linc00942, N^6^‐methyladenosine, RNautophagy

## Abstract

**Background:**

Energy balance has long been known to extend lifespans and inhibit carcinogenesis in multiple species by slowing age‐related epigenetic changes while the underlying mechanisms remain largely unknown. Herein, we found that starvation activated autophagy to remodel the DNA methylation profile by inhibiting DNMT3a expression.

**Methods:**

Illumina Infinium MethylationEPIC BeadChip and dot blot assay were performed to quantify the global DNA methylation level. Protein−RNA interactions were validated through RNA immunoprecipitation and RNA pull‐down assay. In vitro and in vivo experiments were carried out to testify the effect of DNMT3a on chemoresistance.

**Results:**

Autophagy is impaired in chemoresistance which was associated with differential DNA methylation and could be reversed by DNMT3a inhibition. Autophagy activation decreases the expression of DNMT3a mRNA, accompanied with the downregulation of chemoresistance‐related Linc00942. Knockdown of Linc00942 reduces DNMT3a expression and genome‐wide DNA methylation while Linc00942 overexpression increased DNMT3a expression and correlated hypermethylation in cancer cells and primary tumour tissues. Mechanistically, Linc00942 recruits RNA methyltransferase METTL3 to stimulate N6‐methyladenosine (m6A) deposit on DNMT3a transcripts, triggering IGF2BP3/HuR to recognize modified mRNA for reinforced stability. SQSTM1/p62 recruits Linc00942 for autophagic degradation which can be abrogated after autophagy inhibition by p62 knockdown or chloroquine treatment.

**Conclusions:**

Inhibition of autophagy increases Linc00942 expression to promote chemoresistance and autophagy activation or hypomethylating agent decitabine restores chemosensitivity by reducing global DNA methylation. Overall, this study identifies a novel methylation cascade linking impaired RNautophagy to global hypermethylation in chemoresistance, and provides a rationale for repurposing decitabine to overcome chemoresistance in cancer treatment.

## Graphical abstract

- Autophagy remodels global DNA methylation via suppressing DNMT3a expression.
- Impaired autophagy facilitates DNMT3a‐dependent DNA hypermethylation to promote chemoresistance.
- Long non‐coding RNA Linc00942 links DNMT3a mRNA methylation to global DNA hypermethylation.
- Linc00942 promotes METTL3‐mediated m6A modification to stabilize DNMT3a mRNA.
- Autophagy adaptor protein p62/SQSTM1 mediates RNautophagic degradation of Linc00942.
- Demethylation agents holds therapeutic promise against chemotherapy resistance.

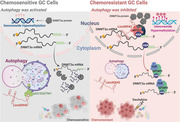

## 1 INTRODUCTION

As an evolutionally conserved catabolic process to clear unnecessary organelles or macromolecules for cellular recycling, autophagy can facilitate cellular adaptation to various intrinsic or environmental stresses, including ageing or nutrient deficiency. It involves the selective transport of damaged or unneeded organelles and macromolecules usually modified by protein ubiquitination to the lysosomes for degradation.^1^ By doing so, autophagy functions as a quality control to enable cellular survival. One of the well‐known inducers of autophagy is nutrient starvation. It was believed that autophagy is important to maintain optimal cellular function during energy metabolism, which has been long‐known to inhibit the progression of ageing and many disorders, such as cancers.^2^, ^3^ p62/SQSTM1 was responsible for recruiting ‘ready‐to‐degradation’ proteins to autophagosome as the adaptor protein. Autophagy could also degrade cancer‐promoting proteins, such as p62, which can activate nuclear factor kappa B or other oncogenic signalling pathways^4^ and has been characterized as a novel RNA‐binding protein^5^, ^6^; however, the physiological and pathological significance of p62 binding to RNA remains unknown. As a result, dysregulation of autophagy contributes to various pathological processes, such as chemoresistance in cancers.^7^, ^8^

The conventional central dogma was commonly described as the coherent transmission of information from DNAs to proteins via RNAs, which involves the consecutive steps of transcription and translation. Nevertheless, the increasing occurrence of unexplained biological phenomenon extended the contents in central dogma, which broadly contains ncRNAs, DNA modifications and post‐transcriptional/translational modifications of RNA/protein that emerged as epigenetic or epitranscriptomic modifications to control multiple cellular processes.^9^, ^10^, ^11^ Meanwhile, epigenetic alterations were pivotal to remodel gene expression profile as another adaptive response to various stresses. Covalent nucleic acid modifications in addition to histone modifications constitute the fundamental component of epigenetic programmes regulating various pathophysiological processes, including cancer development and progression.^9^, ^10^, ^11^ In mammalian cells, DNA modifications mainly refer to DNA methylation which stands for the formation of 5‐methyl‐2′‐deoxycytidine (5‐mC) at the cytosine of CpG dinucleotide, catalysed by DNA methyltransferases (DNMTs), including DNMT1, DNMT3a and DNMT3b.^12^, ^13^, ^14^ DNA methylation is important to maintain genome integrity and regulate the transcription initiation of host genes.

On the other hand, the most prevalent modification of eukaryotic mRNA is N (6)‐methyladenosine (m6A), which plays essential roles in post‐transcriptional RNA processing, transportation, degradation and translation.^15^, ^16^, ^17^ Consistently, dysregulation of RNA modifications is intimately associated with the onset or progression of tumor. Thus, it is momentous to elucidate the detailed dynamic molecular mechanisms of RNA modifications for new diagnostic and therapeutic methods for cancer.^18^, ^19^ m6A modification, a dynamic and reversible bioprocess, is controlled by methyltransferase (known as ‘writers’) consist of methyltransferase‐like 3 (METTL3) complex (METTL3‐METTL14‐WTAP), METTL16, RNA‐binding motif protein 15 (RBM15) and its paralogue RBM15B, zinc finger CCCH‐type containing 13 (ZC3H13), vir‐like m6A methyltransferase‐associated protein (VIRMA or KIAA1429), as well as de‐methyltransferase (known as ‘erasers’) including fat mass and obesity‐related protein (FTO) and alkB homologue 5 (ALKBH5). METTL3‐METTL14‐WTAP complex was the most paramount and YTH domain family proteins, insulin‐like growth factor 2 mRNA‐binding proteins (IGF2BPs) and heterogeneous nuclear ribonucleoprotein family proteins could precisely recognize m6A modified RNAs to accomplish its fate determination.^19^

Together with various histone modifications, DNA and RNA methylations orchestrate a retrograding signalling pathway to regulate gene expression.^20^ For example, DNMT3L recognizes unmethylated H3K4 to induce *de novo* DNA methylation through activating DNMT3A2.^21^ H3K36me2 recruits DNMT3a and shapes the intergenic DNA methylation landscape.^22^ In addition, H3K36me3 participates in m6A modification of nascent RNAs during transcription.^23^ However, the crosstalk between RNA methylation and DNA methylation to modulate gene expression in response to various environmental stresses, such as nutrient starvation or chemotherapy, is yet to be determined.

In this study, we show that long non‐coding RNA Linc00942 connects mRNA methylation to DNA methylation, and autophagy reshapes the global DNA methylation landscape by controlling Linc00942 abundance. Impaired autophagic degradation of Linc00942 upregulates DNMT3a expression at the post‐transcriptional level in an m6A‐dependent manner. Therefore, therapeutic targeting of DNA methylation holds the translational potential to circumvent chemotherapy resistance.

## 2 MATERIALS AND METHODS

### 2.1 Regents, resources, siRNAs, lentivirus and primers

The lentivirus (with GFP fluorescence) for knocking down DNMT3a was purchased from GenePharma and infected with polybrene. Following the selection using puromycin, we obtained the stably DNMT3a knockdown cells. Further information about regents (including antibodies, chemicals, kits of critical Commercial Assays) and resources (including Oligonucleotides, Software/Algorithms and cell lines) used in this article are all listed in Table S1. The detailed sequences of siRNAs and primers are shown in Table S2.

### 2.2 Cell culture and treatments

Human gastric cancer cell lines SGC7901 and BGC823 were purchased from the National Collection of Authenticated Cell Cultures. Multidrug‐resistant cells SGC‐R and BGC‐R were induced from SGC7901 and BGC823 as previously reported,^24^ respectively. Cells were all cultured in RPMI‐1640 or DMEM medium (Invitrogen) supplemented with 10% FBS and 100 U/mL Penicillin‐Streptomycin at 37°C in a 5% CO_2_ atmosphere. All cell lines were handled with Plasmocin® treatment and Plasmocin® prophylactic (InvivoGen, ant‐mpt & ant‐mpp) to eliminate or prevent Mycoplasma contamination.

### 2.3 Animal treatments

All the mice mentioned and used in our research were bred and maintained under defined conditions at the Animal Experiment Center of Zhejiang University (SPF grade). Animals’ solicitude and operations were executed following the Institutional Animal Care and Use Committee and National Institute of Health guidelines. Before performing all the animal experiments, we were approved by the Laboratory Animal Ethics Committee of Sir Run Run Shaw Hospital of Zhejiang University and conformed to the legal mandates. The in vivo tumour growth assays were operated mainly as previously described.^18^ Briefly, 5−8 × 10^6^ PCDH (empty vector) or 942‐OE (transduced with overexpression plasmid) or plvx(empty vector)+shNC, plvx‐942‐OE+shNC, plvx+shDN3a, 942‐OE+shDN3a SGC79001 stably infected cell lines were subcutaneously injected into the dorsal flanks of each mouse (*n* = 8 or 5). After about 1 week, the xenograft mice were divided into two or four groups stochastically. PCDH mice were injected intraperitoneally with DMF or DDP and 942‐OE mice were injected with DMF+DMSO, DDP+DMSO, Decitabine+DMF or DDP+Decitabine, the plvx+shNC, plvx‐942‐OE+shNC, plvx+shDN3a, 942‐OE+shDN3a mice were randomly allocated to eight groups and treated with DMF or DDP (2 or 4 mg/kg), respectively, and measured the tumour volume, weight periodically as indicated. After about 2 weeks, the mice were euthanized, and the tumours were finally dissected and weighed.

### 2.4 Illumina Infinium MethylationEPIC BeadChip and In Silico Analysis

Total DNA from HeLa, SGC7901 and SGC‐R cells with corresponding treatment as indicated was extracted, quantified (> 50 ng/μL) and fullfilled Quality Inspection (OD260/280 = 1.8−2.0 & OD260/280 > 2.0). The perfect prepared DNA was treated with Bisulfite, followed by whole genome amplification and accomplished fragmentation processing. Then, the assessment segues into array (or Chip) hybridization with the well‐modified probe (> 850 000 sites, more than 95% CpG islands and 99% RefSeq genes coverage). The whole project will be completed after thoroughly elution and image acquisition. Finally, In Silico Analysis was implemented including Quality control and rectification for raw data, as well as GO or KEGG enrichment. All procedures mentioned above were all accomplished by Genesky Corporation in Shanghai.

### 2.5 Genome DNA extraction and dot blot for DNA 5‐mC

Total DNA was extracted from cells with specific treatment as indicated and using TIANamp Genomic DNA Kit (Tiangen Biotech). To exclude the possibly contaminated methylated‐RNA, the extracted genome DNA was treated with .1 mg/mL RNase A at 37°C for 1 h. NanoDrop was used to detect the quality (OD260/280 = 1.8−2.0 & OD260/280 > 2.0) of the genomic DNA. Dot blot was operated referring to the procedure in the Bioprotocol website with modification. Briefly, the well‐prepared DNA was denatured at 95°C for 5 min and cooled down immediately on ice for 5 min. DNA was then loaded on the Hybond‐N+ membranes, and air dry for about 5 min. The flat membrane was then performed UV crosslinking (1200 microjoules [x100]; 25–50 s) using Stratalinker 2400 UV Crosslinker. The membrane was washed in a clean washing tray for 5 min at room temperature with gentle shaking to remove the unbound DNA, found by the incubation in 5% non‐fat milk blocking buffer for at least 1 h at room temperature with gentle shaking. The membrane was then incubated with an anti‐5‐mC antibody (1:500 dilution) in 5% non‐fat milk dilution buffer overnight at 4°C with gentle shaking. After washing three times for 5 min each in wash buffer with gentle shaking, the membrane was incubated with goat anti‐mouse IgG‐HRP (1:5000 dilution) for 2 h at room temperature. Finally, the membrane was washed four times for 10 min each before the incubation with moderate ECL Substrate and exposure in darkness. Methylene Blue (.02%) staining was executed as the internal reference of the loading amount for DNA sample.

### 2.6 Cellular viability and apoptosis assay

About 4000−8000 cells were seeded in 96‐well plates and adherent. Chemicals or inhibitors were added to cells as indicated. Cell viability was all measured using the 3‐(4, 5‐dimethylthiazol‐2‐yl)−5‐(3‐carboxymethoxyphenyl)−2‐(4‐sulfophenyl)−2H‐tetrazolium (MTS) standard method at OD 490 nm with BioTek Gen5 system. For apoptosis analysis, the cells with indicated treatment were carefully harvested (digestion using EDTA‐free Trypsin) and made at a ratio of 10^6^ cells/100 μL. Finally, the cells were assessed for apoptosis using an Annexin V‐FITC‐PI dual‐staining kit (556547; BD Biosciences) by flow cytometer.

### 2.7 Autophagy induction and blocking

Autophagy was effectively induced using Earle's balanced salt solution (EBSS) buffer treatment (wash the cells gently with Phosphate Buffered Saline (PBS) before treatment) for 4 or 8 h, or Rapamycin (50 or 200 nM) treatment for 48 h, or Everolimus (25 nM) treatment for 24 h, or Torin 1 (5 μM) treatment for 2 h. Chloroquine (CQ) (50 nM, 24 h) was used to block autophagy. LC3B and p62 were regarded as the marker to make a measurement for autophagy activation or inhibition.

### 2.8 RNA half‐life detection

Gastric cancer cells were transfected with given siRNAs or plasmids at least for 48 h, or incubated with chemicals as indicated. Then, cells in each group were digested and seeded to a 6‐well plate averagely. The adherent cells were treated with Actinomycin D (Act D; 5 μg/mL) to block the synthesis of nascent RNAs. Cells were then harvested to extract total RNA at 0, 2, 4, 6, 8 and 12 h after Act D addition for Reverse Transcription and quantitative PCR. Half‐life curves were plotted on non‐linear fitting and regression (one‐phase decay curve fit) using GraphPad Prism 9.

### 2.9 RNA immunoprecipitation and biotin pull‐down assay

RNA immunoprecipitation (RIP) was performed using Magna RIP™ RNA‐Binding Protein Immunoprecipitation Kit (Millipore). Briefly, at least 1 × 10^7^ cells after the given treatment were lysed in 100 μL RIP Lysis Buffer with Protease Inhibitor and RNase Inhibitor, and immunoprecipitated with antibodies of interest or IgG and protein G magnetic beads overnight at 4°C, followed by six washes in Washing Buffer and protein digestion at 55°C. Total RNA was isolated from the aqueous after digestion and subjected to RT‐PCR analysis for quantification.

For RNA‐protein pull‐down assay, firstly, Streptavidin Magnetic beads (NEB) were pretreated using RNA binding Buffer (50 mmol/L KCl, 1.5 mmol/L MgCl_2_, 10 mmol/L HEPES [PH 7.5], .5% NP40, 2 mmol/L DTT, 1 mmol/L EDTA, 100 U/mL RNase Inhibitor, Protease Inhibitor, 100 μg/mL tRNA and 400 μmol/L Vanady ribonucleoside complexes). RNA−protein complex was formed by incubating 1−2 μg biotin‐labelled probe with cell lysates at 30°C for 30 min. After incubating pretreated Streptavidin Sepharose at room temperature for 50 min, RNA−protein mixture was precipitated and extracted proteins for Western blotting (WB) with 20 μL 6 × Loading buffer after six times strictly washing with RNA washing Buffer (50 mmol/L KCl, 1.5 mmol/L MgCl_2_, 10 mmol/L HEPES [PH 7.5], .5% NP40).

For RNA−RNA pull‐down assay, in brief, cells with indicated treatment were resuspended using lysis buffer (20 mM Tris, pH 7.5, 200 mM NaCl, 2.5 mM MgCl_2_, .05% Igepal, 60U mL‐1, Superase‐In [Takara], 1 mM DTT, protease inhibitors [Roche]). Fifty microlitres lysates were used for input control. Lysates were incubated with prepared Streptavidin Magnetic beads. RNase‐free bovine serum albumin (BSA) and yeast tRNA were used when incubated in blocking lysates at 4°C for 3 h to avoid the non‐specific RNA‐protein binding. Then, washed twice with ice‐cold lysis buffer, three times with the low salt buffer (.1% SDS, 1% Triton X‐100, 2 mM EDTA, 20 mM Tris‐HCl, pH 8.0 and 150 mM NaCl), and once with the high salt buffer (.1% SDS, 1% Triton X‐100, 2 mM EDTA, 20 mM Tris‐HCl, pH 8.0 and 500 mM NaCl). Finally, the bound RNAs were extracted and purified for qRT‐PCR.

### 2.10 Immunologically co‐localization assay

The detection was performed mainly by combining RNA Fluorescence in situ Hybridization (FISH) (probe for Linc00942 is synthesized from LGC Science Ltd) and protein Immunofluorescence (IF) with some modifications. Firstly, cells were seeded in cell climbing sheets of a 12‐well plate and fixed using Fixation Buffer after well adhering, then incubated with the Stellaris RNA FISH probe (LGC) in Hybridization Buffer for at least 4 h at 37°C after using .2% Triton X‐100 for 20 min to permeate the cells. After washing with Wash buffer A, the cells were blocked with 3% BSA for 1 h at room temperature and incubated with anti‐p62/SQSTM1 or anti‐LC3B antibody at 4°C overnight. After washing with PBST three times, the cells were incubated with secondary fluorescent antibodies (the Alexa Fluor 488 goat antirabbit IgG (H+L) secondary antibody) for 1 h at room temperature. before proceeding to imaging. All images were visualized and filmed using an Olympus FV1200 SPECTRAL Laser scanning Confocal Microscope.

### 2.11 Quantification and statistical analysis

Public gene expression data or clinical information were obtained from The Cancer Genome Atlas (TCGA) and GSE62254 cohort in Gene Expression Omnibus (GEO) database. For the processing of microarray data, we downloaded the raw ‘CEL’ files and adopted ‘rma’ method to perform background adjustment and quantile normalization via ‘*affy’* and ‘*simpleaffy’* R packages. All experiments were performed at least in triplicate. Data from qRT‐PCR, MTS, apoptosis and RIP were analysed using Student's *t*‐test. *p* < .05 was considered as statistically significant and ‘*’ represents *p* < .05, ‘**’ represents *p* < .01 and ‘***’ represents *p* < .001. The significance of DNMT3a expression between chemotherapy‐sensitive and chemotherapy‐resistant groups was compared by the Wilcoxon test. Correlation coefficients were calculated by the Pearson and distance correlation analyses.

## 3 RESULTS

### 3.1 Autophagy inhibits DNMT3a expression to remodel global DNA methylation

**FIGURE 1:**
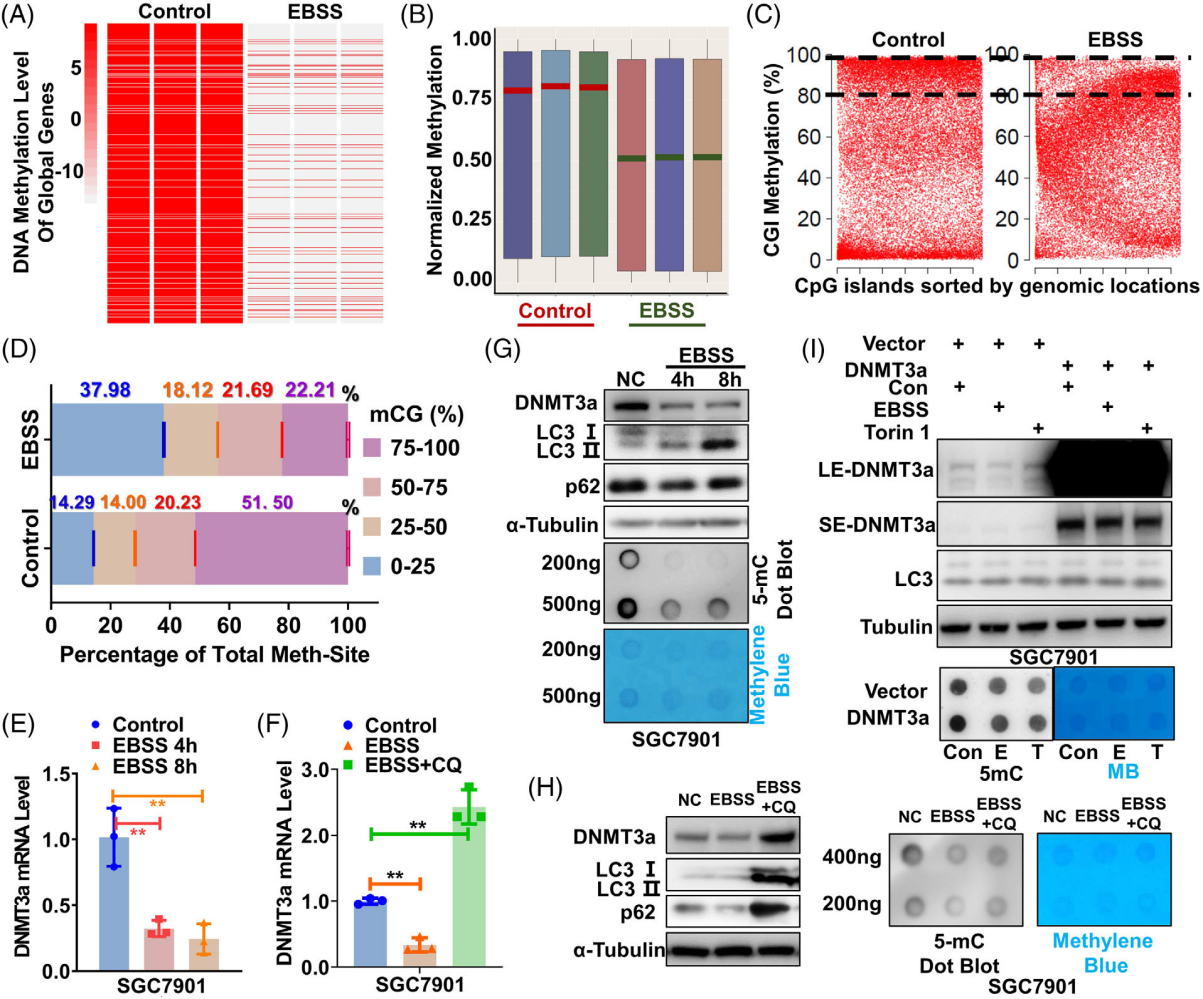
Autophagy inhibits DNMT3a expression to remodel DNA methylation. (A) and (B) The heat map (A) or boxplot (B) of global DNA methylation in SGC7901 cells before and after autophagy activation by EBSS. All methylated genes were ordered randomly. Red or green lines in (B) indicate the medians of three duplicates. Global DNA methylation was detected using MethylationEPIC BeadChip. (C) A scatter plot of CGI methylation before and after autophagy activation. The percentages of CGIs with methylation levels greater than 80% are indicated with dotted lines. (D) A global view of DNA methylation in SGC7901 cells before and after autophagy activation. A cumulative bar plot shows the proportions at four methylation levels (0%−25%, 25%−50%, 50%−75% and 75%−100%). mCG means CG methylation. (E)–(H) The effect of autophagy induction or inhibition by CQ on DNA methyltransferase DNMT3a is determined by Western Blotting (E, F) and qRT‐PCR (G, H). Total DNA 5‐mC level is measured by dot blot. The grey blots indicate DNA methylation levels with methylene blue staining of DNA as the internal reference. (I) Total DNA 5‐mC levels in DNMT3a‐transfected cells before and after autophagy activation using EBSS (Starvation) or Torin 1 (mTOR inhibitor) are determined by dot blot with anti‐5‐mC antibody.

The total 5‐mC level was remarkably reduced in HeLa cells after autophagy induction using EBSS treatment, which could be partly recovered by autophagy inhibitor CQ (Figure S1A,B). Consistently, the global DNA methylation level was  significantly decreased after EBSS treatments in both HeLa and SGC7901 (gastric cancer cell line) cells (Figure 1A‐B and Figure S1C), and the differential methylation sites were independent of specific genome position (Figure S1D). Moreover, the median methylation level and percentage of hypermethylated genes (mCG% = 50−100) overwhelmingly declined after autophagy activation (Figure 1C‐D  and Figure S1E), confirming the relevance of autophagy to global DNA methylation.

Intriguingly, autophagy activation could decrease the expression of DNMT3a mRNA (Figure 1E), which was reversed upon autophagy inhibition (Figure 1F). In contrast, the expression of other DNMTs, such as DNMT3b and DNMT1 mRNA, was not affected by autophagy induction or inactivation in two independent gastric cancer cell lines (SGC7901 and BGC823) (Figure S1F–H). Consistently, DNMT3a protein rather than other DNMTs, and total 5‐mC levels were obviously reduced upon autophagy induction (Figure 1G and Figure S1I,J), which was rescued by autophagy inactivation (Figure 1H). In addition, autophagy‐induced global hypomethylation was not specific to EBSS, as DNMT3a expression and the total 5‐mC level were also reduced after autophagy was activated by other inducers (Figure S1K,L), which can be reversed by the inhibition of autophagy as well (Figure S1M,N). Consistent results were demonstrated using another autophagy inhibitor BafA1 (Figure S1O,P). Finally, DNMT3a overexpression could remarkably rescue the declined DNA methylation level caused by autophagy induction (Figure 1I and Figure S1Q), highlighting the relevance of DNMT3a downregulation to autophagy‐induced global hypomethylation. In brief, autophagy remarkably remodelled DNA methylation profile through reducing DNMT3a expression specifically.

### 3.2 Both DNMT3a expression and global DNA methylation are elevated in chemoresistant cancer cells

**FIGURE 2:**
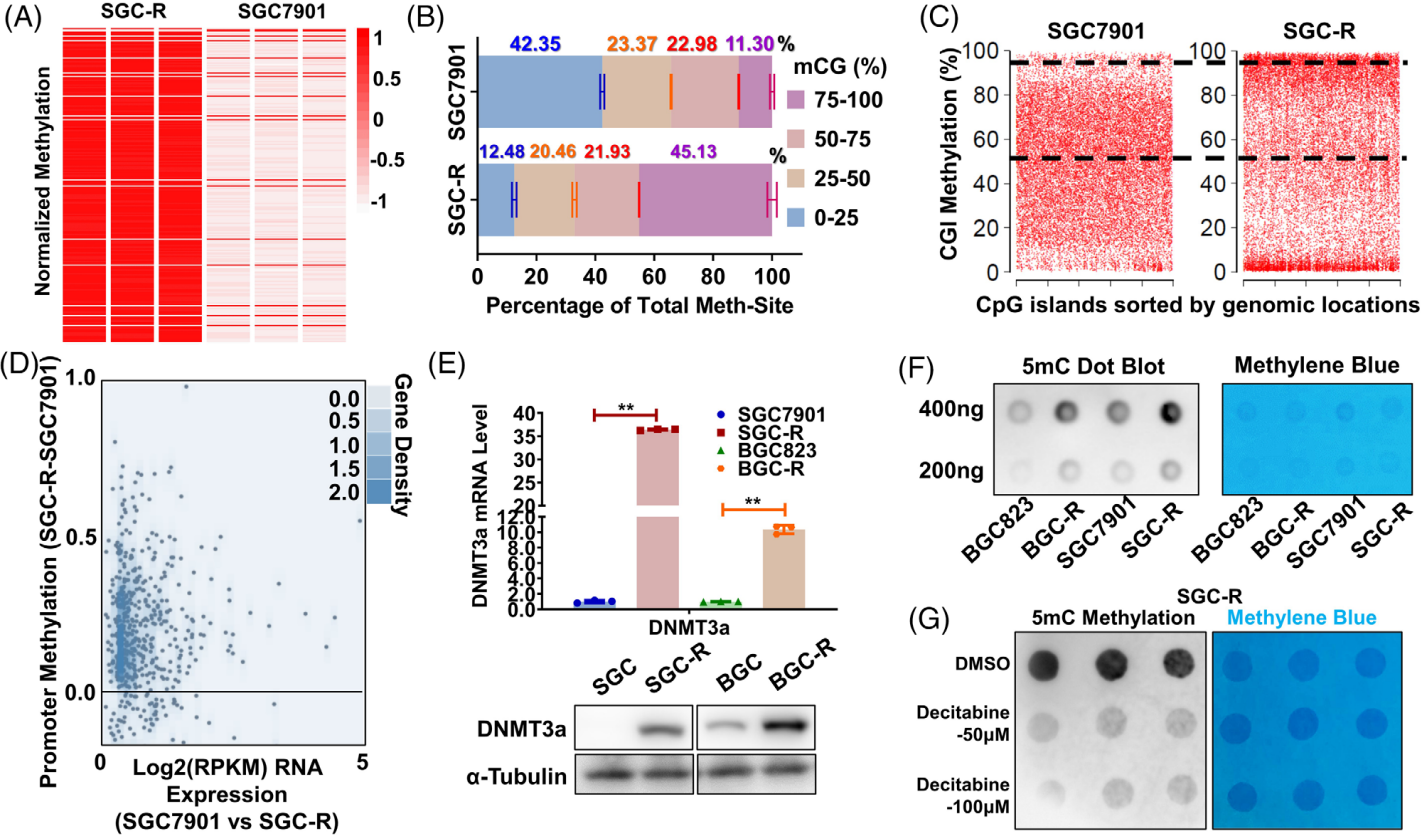
Differential DNA methylation occurs in chemoresistance GC cells. (A) A heat map of global DNA methylation level for chemosensitive (SGC7901) and chemoresistant (SGC‐R) cells using MethylationEPIC BeadChip. All methylated genes were ordered randomly. (B) A global view of DNA methylation in SGC7901 and SGC‐R. A cumulative bar plot shows the proportions at four methylation levels (0%−25%, 25%−50%, 50%−75% and 75%−100%). mCG means CG methylation. (C) The scatter plot of CGI methylation in the Control and EBSS group. The percentages of CGIs with methylation levels greater than 50% are indicated with dotted lines. (D) Plot of the changes of promoter methylation against the average gene expression between SGC7901 and SGC‐R. (E) The expression of DNMT3a in chemosensitive or resistant SGC7901 and BGC823 cells was detected by qRT‐PCR and WB. Data were presented as the mean ± SD, *n* = 3. **p < .01 (Student's *t*‐test). (F) Detection of overall DNA methylation levels by DNA dot blot with antibodies specific for 5‐mC in chemosensitive or resistant SGC7901 and BGC823 cells. The grey values indicate DNA methylation levels and the blue one shows the result of methylene blue staining as DNA internal reference. (G) Dot blot of DNA isolated from DMSO and Decitabine (reduce levels of DNA methylation) treatment in SGC‐R cells is performed to testify the global DNA methylation level.

Autophagy has been reported to play fundamental roles in the development and progression of various cancers, including chemoresistance, hinting that autophagic remodelling of epigenetic programmes might be relevant to cancer progression. Indeed, aberrant autophagy activity was observed in chemoresistant cancer cells (BGC‐R and SGC‐R) which were previously established in‐house,^25^ and a subsequent rise of DNA methylation level was identified compared with parental chemosensitive cancer cells (BGC823 and SGC7901) (Figure 2A). Whereas parental cells displayed a largely normal hypomethylated (mCG% = 0−50) genome, there was an approximately two‐fold increase of hypermethylated (mCG% = 50−100) sites in resistant cells. Of the CpG islands (CGIs), 67% were highly methylated in resistant cells, in contrast to 34% in parental cells (Figure 2B,C). The dysregulation of DNA methylation in resistant cells occurred over a wide range of genomic features, including gene body and 5′UTR (Figure S2A). Besides, we observed a significant trend of gain in aberrant DNA methylation on promoter regions of downregulated genes in resistant cells (Figure 2D), confirming the transcriptional inactivation of hypermethylated promoters. Many genes that were important to autophagy or drug metabolism were dysregulated in resistant cells (Figure S2B). Afterwards, both mRNA and protein of DNMT3a but not DNMT3b or DNMT1 were overwhelmingly upregulated in two independent resistant cell lines SGC‐R and BGC‐R compared to parental cells, respectively (Figure 2E and Figure S2C,D). Consequently, the total 5‐mC level was exceedingly higher in resistant cells than in sensitive ones (Figure 2F). The DNA methylation inhibitor decitabine could dramatically decrease the 5‐mC level in both SGC‐R and BGC‐R cells (Figure 2G and Figure S2E). Taken together, both DNMT3a and global DNA methylation were upregulated in chemoresistant cancer cells.

### 3.3 Increased DNMT3a expression contributed to chemoresistance

**FIGURE 3:**
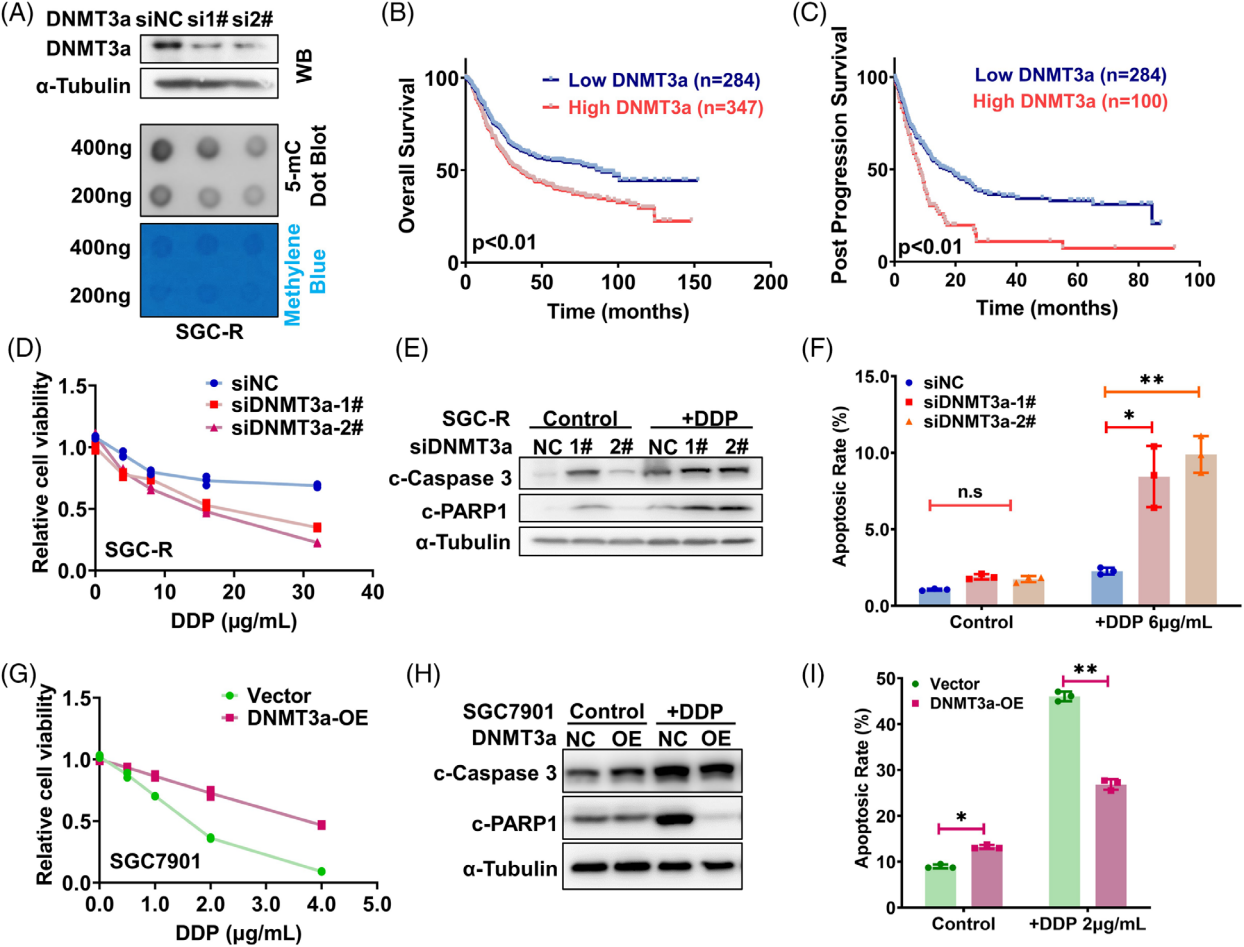
DNMT3a induces differential DNA methylation in chemoresistance cells. (A) DNMT3a was knocked down in SGC‐R and the efficiency is assessed by WB. Dot blot of DNA isolated from indicated groups is operated to identify global DNA methylation level. (B, C) The Kaplan–Meier curve analysis on the impact of DNMT3a expression on overall survival (B) and post progression survival (C). *p* Value was calculated by the Log Rank test. (D) Effect of DNMT3a knockdown in SGC‐R on the viability of resistant cells with or without DDP treatment for 24 h was detected using MTS assay. Experiments were all repeated three times and the representative data were shown. (E, F) SGC‐R cells transfected with negative control (siNC) or DNMT3a siRNAs were treated with or without DDP (5 μg/mL) for 24 h and dynamic apoptosis rate was measured using WB (E) as well as flow cytometry (F). (G) Effect of DNMT3a overexpression in SGC7901 on the viability of sensitive cells with or without DDP treatment for 24 h was detected using MTS assay. (H, I) SGC7901 cells transfected with empty or DNMT3a overexpression vector were treated with or without DDP (2 μg/mL) for 24 h and dynamic apoptosis rate was measured using WB (H) as well as flow cytometry (I).

To demonstrate the relevance of dysregulated DNMT3a to chemoresistance, we firstly validated the decrease of 5‐mC level in resistant cells when DNMT3a was knocked down (Figure 3A). Accordingly, clinical data from TCGA database suggested that higher DNMT3a expression was correlated to shorter overall survival and post progression survival in gastric cancer patients (Figure 3B,C), strongly indicating that upregulated DNMT3a may mediate chemoresistance in gastric cancer. Indeed, knocking down DNMT3a in resistant cells exceedingly reversed chemoresistance since drug‐induced viability inhibition and apoptosis were greatly enhanced (Figure 3D–F and Figure S3A–D). On the contrary, the expression of ectopic DNMT3a dramatically attenuated viability inhibition and apoptosis after drug treatment in sensitive cells (Figure 3G–I and Figure S3E–H). Additionally, we found a significantly higher expression of DNMT3a in the chemotherapy‐resistant (Non‐response) group compared to the chemotherapy‐sensitive group (Response) in the GSE62254 cohort of gastric cancer patients (Figure S3I). Taken together, increased DNMT3a expression contributed to chemoresistance.

### 3.4 Linc00942 increased the 5‐mC level through upregulating DNMT3a expression

**FIGURE 4:**
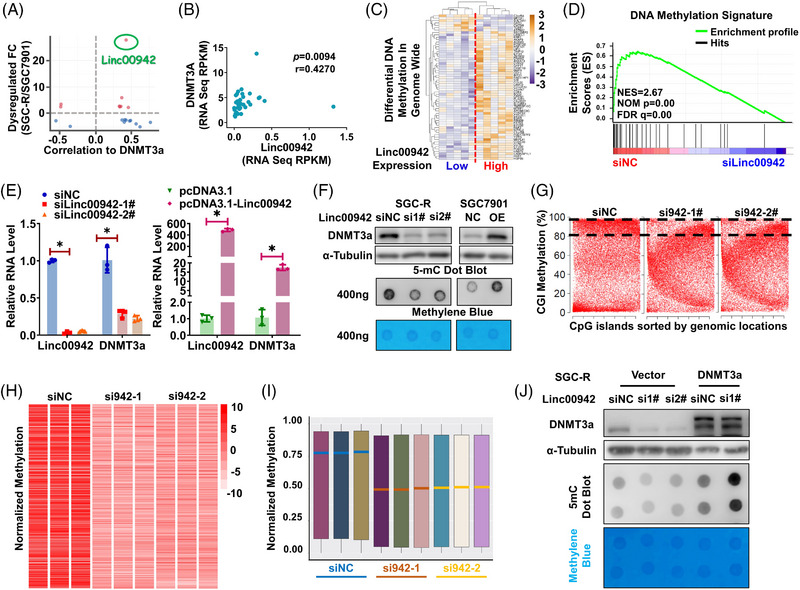
Linc00942 could increase DNA methylation level by upregulating DNMT3a expression. (A) The cross‐scatter plot of dysregulated genes in SGC7901 and SGC‐R with DNMT3a correlated genes which analysed in TCGA database. (B) Scatter plot of DNMT3a and Linc00942 expression (correlation coefficient, *r* = .4270; *p* = .0094). (The normalized expression data were downloaded in TCGA database.) (C) The heatmap of significantly differential DNA methylation genes in Linc00942 low and highly expressed samples. (D) Gene Set Enrichment Analysis (GSEA) of dysregulated genes when Linc00942 is knocked down using siRNA. Red indicates siNC; blue indicates siLinc00942. (E, F) Expression of DNMT3a in SGC‐R or SGC7901 cells transfected with Linc00942 siRNAs or overexpression vector, respectively, are analysed by qRT‐PCR (E) and WB (F). Dot blot assay is performed to testify global DNA methylation level. (G) The scatter plot of CGI methylation in siNC and siLinc00942 groups. The percentages of CGIs with methylation levels greater than 80% are indicated with dotted lines. (H, I) A heat map or (I) boxplot of global DNA methylation level for siNC and siLinc00942 treatment groups in SGC‐R cell using MethylationEPIC BeadChip. All methylated genes were ordered randomly. Red or green lines in (I) indicate the medians of three duplicates in the indicated group. (J) Effect of DNMT3a overexpression on global DNA methylation in SGC‐R cells before and after Linc00942 KD was determined by WB and dot blot assay.

To clarify the mechanism for the dysregulation of DNMT3a mRNA, we integrated DNMT3a mRNA correlated genes from TCGA datasets. Long non‐coding RNA Linc00942 was identified as one of the most relevant genes to DNMT3a mRNA expression (Figure 4A and Figure S4A), and its expression was significantly correlated with DNMT3a mRNA (Figure 4B). Besides, we analysed DNA methylation data from TCGA and conclusively noted that genome‐wide DNA methylation levels in Linc00942 high‐expression datasets seemed significantly higher than in the low‐expression ones (Figure 4C). Gene Set Enrichment Analysis based on expression profiling before and after Linc00942 knockdown (KD) indicated that DNA methylation level was reduced in Linc00942 KD cells (Figure 4D). Furthermore, both the mRNA and protein abundance of DNMT3a, rather than DNMT3b or DNMT1, was positively affected by Linc00942 (Figure 4E,F and Figure S4B–D). Coincidently, we also found a remarkable upregulation of Linc00942 in the chemotherapy‐resistant group from the same cohort (GSE62254), and a positive correlation was found between Linc00942 and DNMT3a mRNA level (Figure S4G,H). Consequently, the total 5‐mC level possessed changes corresponding to DNMT3a expression (Figure 4F and Figure S4E). What is more, DNA Methylation Array further confirmed that global DNA methylation tends to dramatically decline and the percentage of genes with high‐level methylation obviously dropped after knocking down Linc00942 (Figure 4G–I and Figure S4F). Subsequently, DNMT3a overexpression could partly rescue the decline of global DNA methylation caused by Linc00942 KD (Figure 4J). To sum up, Linc00942 increased the 5‐mC level through upregulating DNMT3a expression.

### 3.5 Linc00942 stabilizes DNMT3a mRNA by recruiting METTL3 to facilitate its m6A modification

**FIGURE 5:**
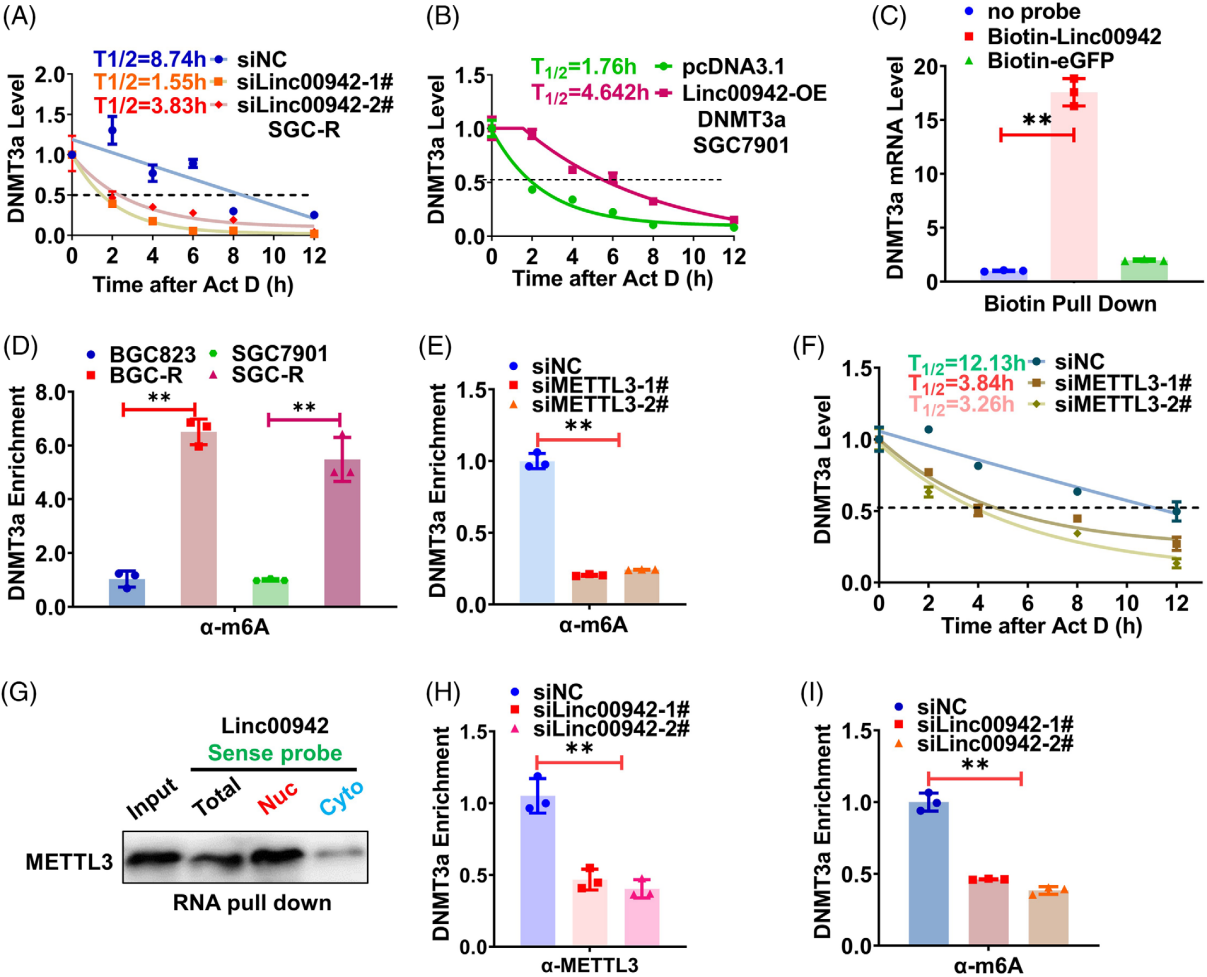
Linc00942 stabilizes DNMT3a mRNA by recruiting METTL3 to facilitate its m6A modification. (A, B) DNMT3a mRNA half‐life before and after knocking down Linc00942 levels in SGC‐R (A) or overexpressing Linc00942 in SGC7901 (B). Total RNAs from cells transfected with indicated siRNAs or plasmids are collected after Act D treatment for 0, 2, 4, 6, 8 and 12 h. (C) eGFP negative or sense probe of Linc00942 was biotin‐labelled and used to pull‐down DNMT3a mRNA. qRT‐PCR is operated to detect the mRNA enrichment. (D) The m6A modification level of DNMT3a mRNA in chemosensitive and chemoresistant cells is assessed by meRIP assay combined with qRT‐PCR. The enrichment fold changes are normalized to Input. (E) The m6A modification level of DNMT3a mRNA before and after METTL3 knockdown is assessed by meRIP assay combined with qRT‐PCR. (F) Changes of DNMT3a mRNA half‐life in SGC‐R before and after knocking down METTL3. (G) The binding of METTL3 to Linc00942 in the cytoplasm and nuclear component was determined by RNA pull‐down assay. (H, I) The effect of Linc00942 knockdown on METTL3 binding (H) or m6A modification (I) of DNMT3a mRNA was assessed by RIP assay.

In an effort to explore the effect of Linc00942 on DNMT3a mRNA expression, we discovered that the stability of DNMT3a mRNA was changed with altering Linc00942 level. The half‐life of DNMT3a mRNA tends to decline or prolong upon knocking down or overexpressing Linc00942, respectively (Figure 5A,B and Figure S5A,B). Considering the strong positive correlation, we assumed a direct interaction between Linc00942 and DNMT3a mRNA. Indeed, the biotin pull‐down assay verifies that a multitude truncation of Linc00942 segments could bind to DNMT3a mRNA (Figure 5C and Figure S5C,D).

In light of the potential role of m6A modification in influencing RNA stability, we performed meRIP‐seq to demonstrate that m6A modification existed in the junction of exon and 3′UTR of DNMT3a mRNA (Figure S5E), which was further followed by RIP‐qPCR verification (Figure S5F). In fact, the m6A modification level of DNMT3a mRNA was more dominant in resistant cells that express higher level DNMT3a as shown above (Figure 5D). We screened the main methyltransferase and found METTL3 methtyltransferase complex seemed more momentous in writing m6A modification to DNMT3a mRNA since knocking down of METTL3 or METTL14 greatly reduced the m6A modification (Figure 5E and Figure S5G). However, METTL14 could not directly bind Linc00942 or DNMT3a to monitor its capacity to interact with METTL3, nor does not influence DNMT3a protein level, ultimately, METTL3 was remarkably selected as the effect writer and recruited by Linc00942 to DNMT3a mRNA (Figure S5H–L). Consequently, DNMT3a mRNA stability was decreased upon METTL3 KD (Figure 5F). Interestingly, Linc00942 interacted with METTL3 in the nucleus to make the writer recruitment possible as a scaffold (Figure 5G). Upon Linc00942 KD, both the interaction of METTL3 (not its protein level, see Figure S5M,N) with DNMT3a mRNA and its m6A modification were significantly impaired (Figure 5H,I). Taken together, Linc00942 stabilizes DNMT3a mRNA by recruiting METTL3 to facilitate m6A modification.

### 3.6 Linc00942 enhanced IGF2BP3/HuR‐dependent stabilization of DNMT3a mRNA

**FIGURE 6:**
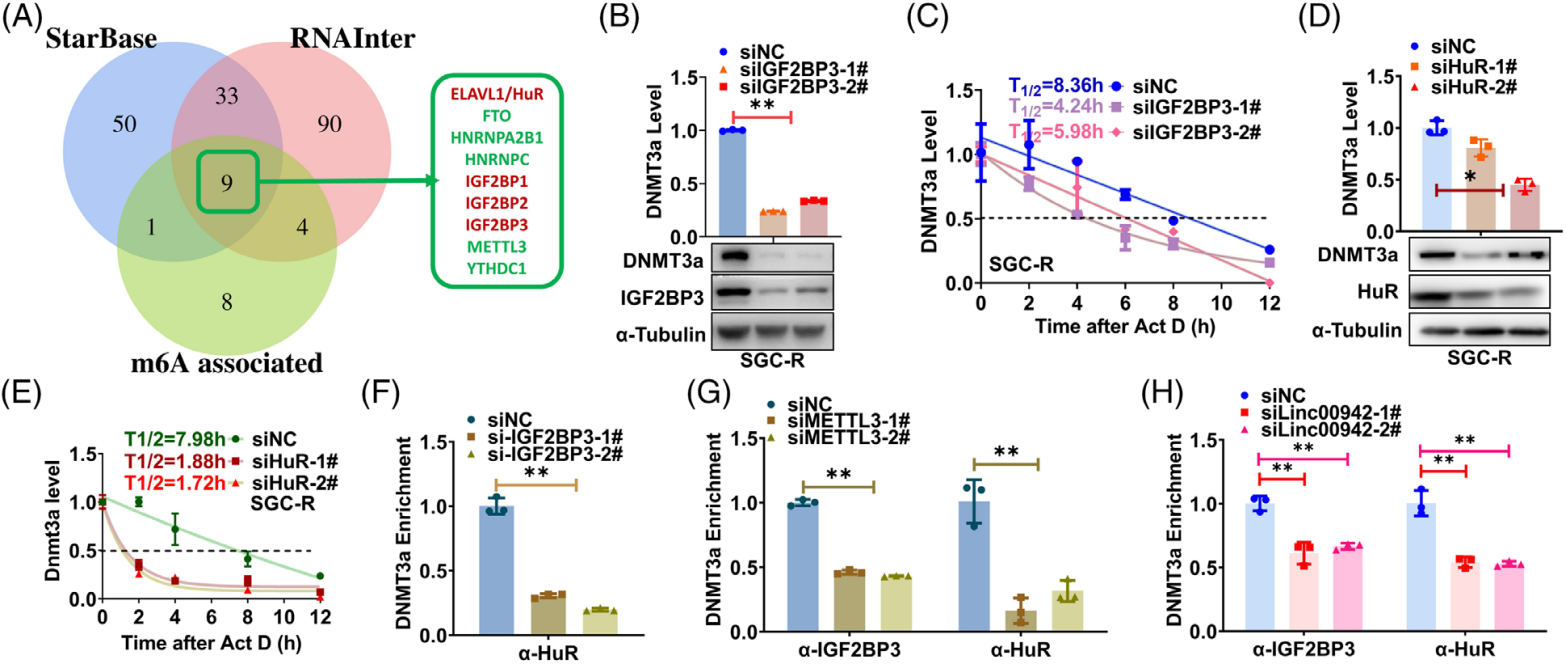
Linc00942 enhanced IGF2BP3/HuR‐dependent stabilization of DNMT3a mRNA. (A) Screening of potential m6A‐associated RBPs interacting with DNMT3a mRNA using StarBase and RNAInter database. (B, C) The effect of IGF2BP3 KD on DNMT3a expression (B) and mRNA stability (C) in SGC‐R cells. (D, E) The effect of HuR KD on DNMT3a expression (D) and mRNA stability (E) in SGC‐R cells. (F) The effect of IGF2BP3 KD on the interaction of DNMT3a mRNA with HuR was assessed by RIP assay. (G) The effect of METTL3 KD on the interaction of DNMT3a mRNA with IGF2BP3 or HuR was assessed by RIP assay. (H) The effect of Linc00942 KD on the interaction of DNMT3a mRNA with IGF2BP3 or HuR was assessed by RIP assay.

To annotate which RNA binding proteins might be relevant to affect the stability of DNMT3a, we thoroughly screened StarBase and RNA Interactome Database to discover the common m6A‐associated RBPs which could potentially interact with DNMT3a mRNA. HuR/ELAV1 and IGF2BP family proteins turned out to be the most noteworthy on account of their feasible function in influencing RNA stability through distinguishing m6A modification RNAs (Figure 6A and Figure S6A,B).^26^ In addition, IGF2BP3 rather than IGF2BP1 nor IGF2BP2 was significantly upregulated in chemoresistant cells and positively modulate DNMT3a mRNA or protein level (Figure 6B and Figure S6C–G), as well as the stability of DNMT3a mRNA (Figure 6C and Figure S6G). Meanwhile, HuR also influenced DNMT3a mRNA expression and stability in the same way (Figure 6D,E and Figure S6H,I). While both IGF2BP3 and HuR could bind to DNMT3a mRNA (Figure S6J,K), IGF2BP3 KD significantly attenuated the binding of HuR to DNMT3a mRNA (Figure 6F), indicating that IGF2BP3 might recruit HuR to stabilize DNMT3a mRNA as previously reported.^26^ Furthermore, the interaction of HuR or IGF2BP3 with DNMT3a mRNA was impaired once the m6A modification was reduced by the KD of either METTL3 or Linc00942 (Figure 6G,H). Collectively, Linc00942 stabilizes DNMT3a mRNA by stimulating METTL3‐dependent m6A modification for IGF2BP3/HuR recognition.

### 3.7 p62 mediated RNautophagic degradation of Linc00942

**FIGURE 7:**
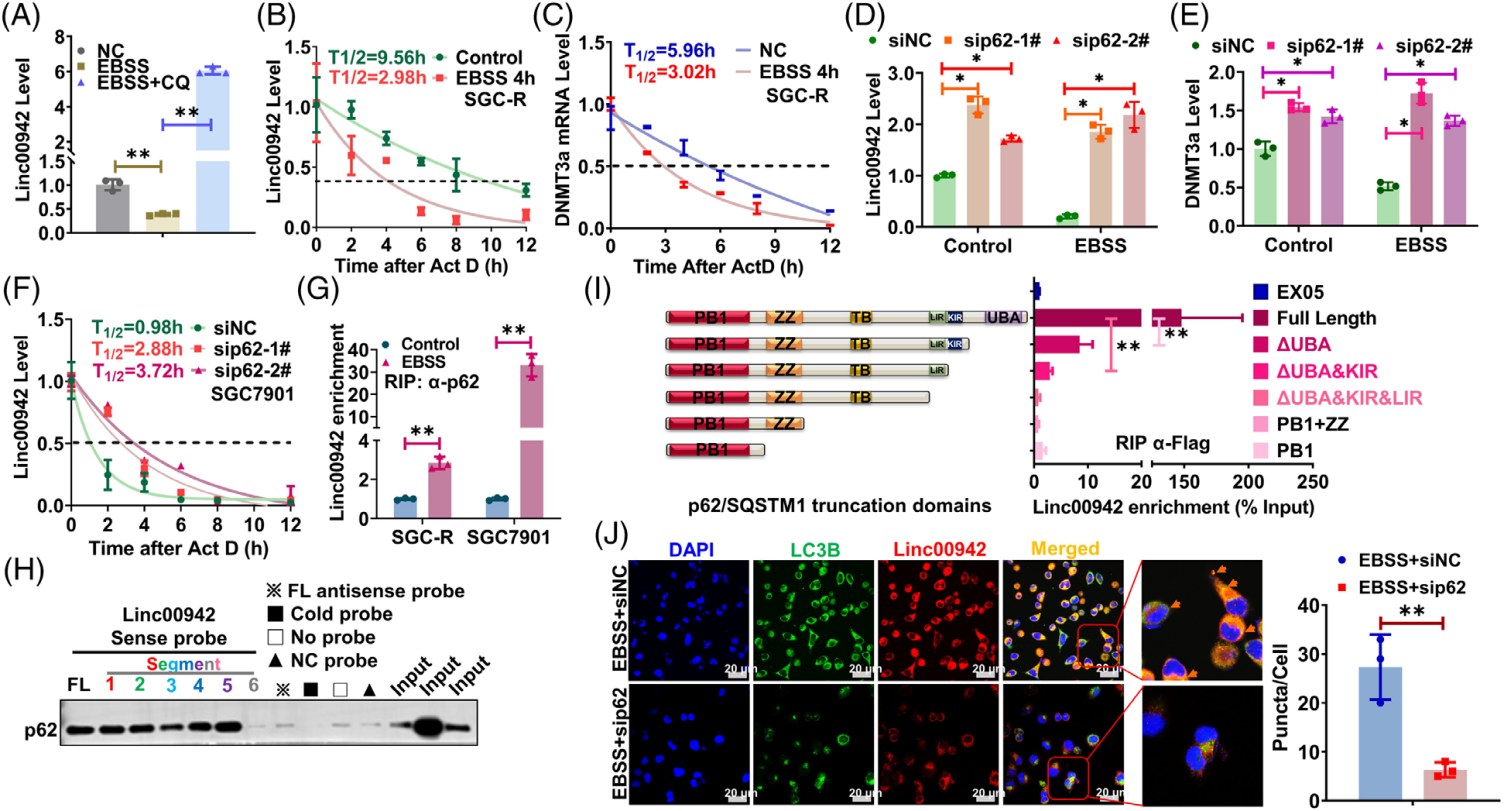
p62 mediated RNautophagic degradation of Linc00942. (A) The effect of autophagy induction or inhibition on Linc00942 level in SGC7901 is determined by qRT‐PCR. (B) The half‐life of Linc00942 in SGC‐R cells before and after EBSS treatment was determined by qRT‐PCR. Half‐life curves were plotted on non‐linear fitting and regression (one‐phase decay curve fit). (C) The half‐life of DNMT3a mRNA in SGC‐R cells treated as indicated was determined by RT‐PCR. (D) The effect of p62 KD on Linc00942 expression before and after EBSS treatment is determined by qRT‐PCR. (E) The effect of p62 KD on DNMT3a mRNA before and after EBSS treatment is determined by qRT‐PCR. (F) The half‐life of Linc00942 in SGC7901 cells before and after p62 KD was determined by RT‐PCR. (G) The effect of autophagy activation on the interaction of p62 with Linc00942 was analysed by RIP assay. (H) The interaction of p62 with various Linc00942 segments was analysed by RNA pull‐down assay. (I) The interaction of Linc00942 with various p62 truncations was analysed by RIP assay. (J) The colocalization of Linc00942 with LC3B in SGC‐R cells treated as indicated was analysed by combined FISH and IF assay (original magnification, 100X). Scale bar: 20 μm.

It has been reported that deregulated autophagy contributed to chemoresistance^25^ and a multitude of RNAs could be degraded in autophagy‐dependent manner (RNautophagy), which was mediated by adaptor proteins, such as p62^24^ and LAMP2C.^27^ Indeed, the abundance of Linc00942 was notably dropped once autophagy was activated, which was significantly reversed after autophagy inhibition (Figure 7A and Figure S7A). As a result, DNMT3a expression as well as the global 5‐mC level was reduced after autophagy activation, which could be rescued by Linc00942 overexpression (Figure S7B–E). Autophagy activation remarkably attenuated the stability of Linc00942 and DNMT3a mRNA, shortening the half‐life from 9.56 to 2.98 h and 5.96 to 3.02 h, respectively (Figure 7B,C). Considering the effect mediator, we choose six familiar adaptor proteins to further screening and p62 was the most dominant one to accelerate RNA decay (Figure 7D,E and Figure S7F). In line with the effect of Linc00942 on DNMT3a, DNMT3a expression was indeed downregulated after autophagy activation which was rescued by p62 KD (Figure 7E and Figure S7G). The foreshortened half‐life of Linc00942 could be rescued by p62 KD (Figure 7F). In chemosensitive cells that have low Linc00942 expression level, p62 depletion also prolonged Linc00942 half‐life from .98 to 2.88 h or 3.72 h (Figure 7F).

Additionally, p62 could interact with Linc00942 which was notably compromised in chemoresistant cells but significantly enhanced after autophagy activation (Figure 7E and Figure S7G,H). Intriguingly, p62 was inclined to bind quite a range of Linc00942 segments (Figure 7H and Figure S5C). On the other hand, the UBA and KIR domain of p62 may be indispensable for interacting with Linc00942 (Figure 7I). Meanwhile, Linc00942 was co‐localized with p62 as well as LC3B puncta only in the presence of p62 upon inducing autophagy (Figure 7J and Figure S7J), indicating that p62 recruits Linc00942 for autophagic degradation. Consistently, p62 overexpression promoted Linc00942 degradation and subsequent downregulation of DNMT3a expression as well as global 5‐mC level, which was reversed by Linc00942 overexpression (Figure S7K,L). Overall, these data implicated that p62 mediated RNautophagic degradation of Linc00942.

### 3.8 Autophagic regulation of DNA methylation is relevant to chemoresistance

**FIGURE 8:**
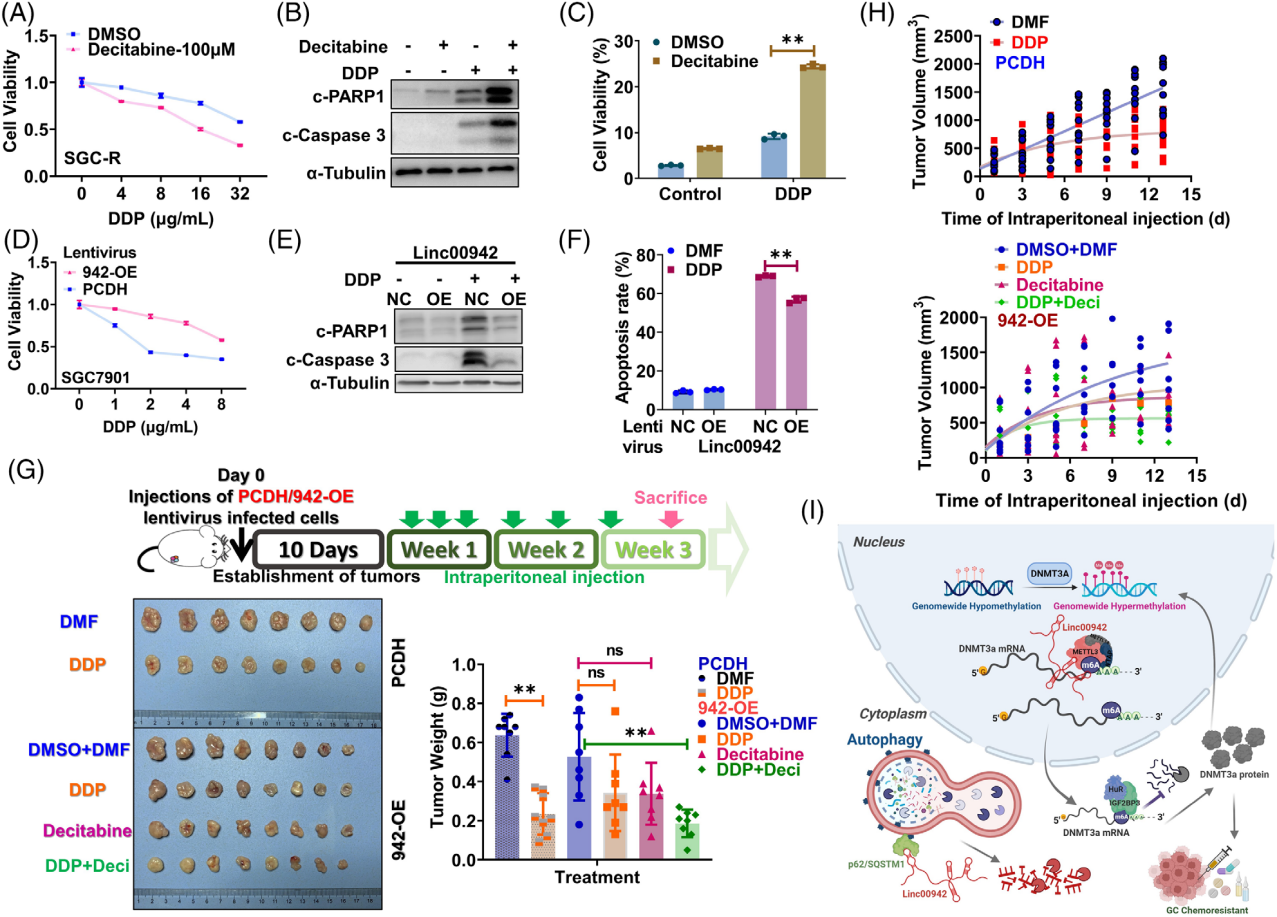
Autophagic regulation of DNA methylation is relevant to chemoresistance. (A) The effect of Decitabine on viability of SGC‐R cells with or without DDP treatment for 24 h was determined by MTS assay. (B, C) The effect of Decitabine on the apoptosis of SGC‐R cells with or without DDP treatment for 24 h was assessed by WB (B) and Flow Cytometry (C). (D–F) The effect of Linc00942 overexpressed (942‐OE) on SGC7901 cells with or without DDP treatment for 24 h was detected by MTS assay (D), WB (E) and Flow Cytometry (F). (G) and (H) The effect of DDP and/or Decitabine on tumour growth of SGC7901 cells with or without Linc00942 overexpression was determined by xenografts assay in nude mice (*n* = 8/group). The dose and injection cycle of DDP or Decitabine is shown in the illustration. Representative photographs of tumours excised from the mice and the terminal weight on day 18 are shown in G. (I) Working model: Autophagy is impaired to degrade Linc00942 in chemoresistant cells. Linc00942 recruits METTL3 to mediate m6A modification in DNMT3a mRNA which is recognized by IGF2BP3/HuR to enhance its stability. Therefore, the upregulation of Linc00942 increased DNMT3a expression to promote chemoresistance.

As Linc00942 upregulation contributed to chemoresistance,^28^ activation of autophagy by mTOR inhibition overcame chemoresistance, which was reversed by ectopic expression of Linc00942 (Figure S8A). In contrast, autophagy inhibition conferred chemoresistance only in the presence of Linc00942 (Figure S8B). Similarly, DNA methylation inhibitor decitabine reversed chemoresistance by inducing more apoptosis and viability inhibition (Figure 8A–C and Figure S8C–F), confirming the relevance of Linc00942‐regulated DNA hypermethylation to chemoresistance. Indeed, Linc00942 overexpression conferred chemoresistance both in vitro and in vivo (Figure 8D–H). Both drug‐induced viability inhibition and apoptosis were compromised upon Linc00942 overexpression (Figure 8D–F). Chemotherapeutic drug cisplatin significantly inhibited the xenograft growth of SGC7901 cells but not SGC7901 cells with Linc00942 overexpression, while the combination of Decitabine and DDP possessed favourable therapeutic efficacy in vivo (Figure 8G,H). Furthermore, the reduced level of DNMT3a (shDNMT3a+942‐OE Group) could prominently reverse the chemoresistance of Linc00942 high expression cells (shNC+942‐OE Group) in vivo. Indicated stable cell line was constructed starting afresh (Figure S8G,H) and injected into xenograft mice (Figure S8I,J). In short, decitabine reversed the chemoresistance conferred by DNA hypermethylation resulting from impaired RNautophagic degradation of DNMT3a mRNA.

## 4 DISCUSSION

Precision medicine based on the analysis of genetic codes has significantly improved the prevention and treatment of human cancers. Different from genetic codes, epigenetic codes change dynamically with internal or environmental signals to constitute a retrograde signalling pathway, representing a new challenge in the precision diagnosis and treatment of human cancers.^20^, ^29^ DNA methylation was the most important covalent DNA modification in mammalian cells. While DNMTs including DNMT3a and DNMT3b for the *de novo* DNA methylation and DNMT1 responsible for maintenance methylation play critical roles in DNA methylation, DNA demethylation is relatively complex and involves a cascade reaction involving multiple biochemical enzymes, such as ten‐eleven translocation/thymine DNA glycosylase (Tet/TDG).^30^, ^31^, ^32^, ^33^ However, there is still little knowledge about the remodelling of global DNA methylation in response to a dynamic environment. Histone methylation, including H3K36me2/3, can recruit DNMTs to promote DNA methylation in specific regions, thus forming a methylation cascade in epigenetic regulation.^13^, ^18^, ^22^, ^34^ In the present study, we revealed a new methylation cascade to regulate epigenetic profile in response to environmental stresses (Figure 8I). Upon autophagy activation by stresses like starvation, global DNA methylation was reduced since DNMT3a expression was downregulated due to decreased mRNA stability. These data bring some controversy, as promoting autophagy induction would lead to a decrease of DNMT3a, since it is reported by the literature that increased expression of DNMT3a upon different autophagy stimulus.^35^, ^36^ We think it might be the different treatment strategies that result in this controversy. Since previous reports determined DNMT3a mRNA expression after 1−4 weeks recovery with normal culture conditions following short autophagy induction,^35^ we measured DNMT3a expression immediately after autophagy induction or blockage. The timepoints and recovery treatment will be the main causation of the contradiction. It has been well‐known that DNMTs, including DNMT3a, were upregulated in many disorders, such as cancers. Transcriptional activation or inhibition of proteasomal degradation are the major cause for DNMTs upregulation and much less is known about the regulation of DNMTs mRNA stability.^18^, ^37^, ^38^ Interestingly, other DNMTs, such as DNMT3b, could also be upregulated through HuR and lncRNA‐dependent stabilization of its mRNA,^39^ echoing the relevance of ncRNA‐mediated regulation of mRNA stability to epigenetic reprogramming in rapid response to the dynamic environment.

As recently recognized, m6A modification is critical for the fate determination of host mRNAs.^40^, ^41^ However, the mechanism about the formation of m6A modification was much less studied. Interestingly, histone modification not only regulates DNA methylation but also affects m6A modification. For instance, H3K36me3 can be recognized by the methyltransferase METTL14 to stimulate the occurrence of m6A modification during gene transcription.^23^ Unfortunately, it remains largely unknown how particular mRNAs were specifically targeted by methyltransferases for m6A modification. In this study, we found that Linc00942 recruits METTL3 to DNMT3a mRNA for facilitating its m6A modification, which was in turn recognized by IGF2BP3 to promote HuR‐dependent stabilization of DTMT3a mRNA. Non‐coding RNAs, such as lncRNAs, have been found to play a wide range of roles through the interaction with proteins and various RNAs. For example, Linc00942 could notably reduce GCLC mRNA levels to maintain glutathione redox homeostasis.^42^ Interestingly, Linc00942 could directly recruit METTL14 protein, the core component of METTL3 methyltransferase complex, to promote the m6A modification, thereby effectively enhancing the stability of downstream targets, including CXCR4 and CYP1B1 mRNAs.^43^ In line with this finding, herein, we further confirmed that upregulated Linc00942 recruits METTL3 methyltransferase complex to DNMT3a mRNA for m6A modification. Dissimilarly, we showed the predominance of METTL3 in this study and Linc00942 could not influence the protein level of METTL3, as well as METTL14 (Figure S5N). As ncRNAs could pair with target mRNAs, thus conferring the specificity of m6A modification. However, the molecular basis for the interaction of Linc00942 with METTL3 methyltransferase complex warrants further investigation. It would be interesting to know whether METTL3 interacts with Linc00942 directly or through METTL14.

Importantly, the abundance of Linc00942 alters with the activation or inhibition of autophagy, which was believed to play an important role in the regulation of metabolic homeostasis. Autophagy is generally considered to digest protein substrates or organelles as an adaptive response to various stresses mainly nutrient deficiency.^44^, ^45^ In addition, nucleic acids, either DNAs or RNAs, can be directly taken up and degraded by lysosomes in an ATP‐dependent manner, termed RNautophagy/DNautophagy, respectively.^46^, ^47^ The key adaptor protein p62 responsible for recruiting degradable proteins to the autophagosome can bind to RNAs and promote RNautophagic degradation of ncRNAs, such as ARHGAP5‐AS1^24^, thus extending the function of p62 in the regulation of RNA homeostasis. Coincidentally, p62 was recently characterized as a novel RNA‐binding protein, despite lacking a classical RNA‐binding domain.^5^, ^48^ It might be possible that p62 interacts with RNAs to be degraded through Rnautophagy depending on the secondary structure of target RNAs with potential modifications to be identified. Nevertheless, autophagy reshapes the global DNA methylation profile by affecting the amount of Linc00942 to alter DNMT3a expression in an m6A‐dependent manner, thus forming a methylation cascade from RNA methylation to DNA methylation in retrograde signalling pathways. Upon autophagy activation by nutrient starvation or mTOR inhibition, Linc00942 was recruited by p62 for autophagic degradation so that the m6A modification of DNMT3a mRNA was reduced and DNMT3a expression was downregulated, accompanied with a decrease of global DNA methylation. Though how autophagy activation augments Linc00942 for RNautophagic degradation via p62 needs further investigation, this newly defined methylation cascade could act in complement with another cascade from histone methylation to DNA methylation to reform the DNA methylation landscape in response to the dynamic environment, which confers the adaption plasticity, such as chemoresistance to the host cancer cells.

In conclusion, autophagy adaptor protein p62 mediates RNautophagic degradation of Linc00942, which links mRNA methylation to DNA methylation by facilitating METTL3‐mediated m6A modification to stabilize DNMT3a mRNA. Impaired autophagy stimulates DNMT3a‐dependent DNA hypermethylation to promote chemoresistance, confirming the therapeutic promise of demethylation agents against chemotherapy resistance (Figure 8I). Clinical trials have approved several RNA‐based medications, and it is possible to improve the efficiency of RNA drug delivery through the combination of chemical modification and conjugation of RNA with nanocarrier systems. Additionally, our study broadened the application of Decitabine in the therapy of solid tumours. Unfortunately, it was quite difficult to collect tissue sample for immunohistochemistry (IHC) analysis before and after chemotherapy. To sum up, response to treatment is one of the most powerful prognostic markers of cancer and we aim at validating the Linc00942−DNMT3a−DNA methylation axis as the prospective factor for evaluating chemoresistant gastric cancer.

## AUTHOR CONTRIBUTIONS

L. Zhu, Y. Zhu, F. Li, Y. Meng, H. Wang, L. Feng and W. Xu performed experiments. L. Zhu, Y. Zhu, Y. Meng, X. Wang and H. Jin analysed data. L. Zhu, Y. Zhu, F. Li, H. Wang, L. Feng and H. Jin wrote the manuscript. L. Zhu, L. Feng and H. Jin designed the study.

## CONFLICT OF INTEREST STATEMENT

No potential conflict of interest was reported by the author(s).

## Supporting information

Supporting information

Supporting information

Supporting information

## Data Availability

All the Global DNA Methylation Array data could be checked in GEO, with accession number ‘GSE210596’ (You may view complete raw data at: https://www.ncbi.nlm.nih.gov/geo/query/acc.cgi?acc=GSE210596, with the secure token: efwvaowibheftgn). The RNA sequencing data in whole transcriptome as for knocking down Linc00942 could be download in GEO, with accession number 'GSE212184' (you may view complete raw data at: http://www.ncbi.nlm.nih.gov/geo/query/acc.cgi?acc=GSE212184, with the secure token: ydmdciqyzzgbpaz) The global lncRNA array data used in Figure S4A were already uploaded in GEO previously. You can view the data with accession number ‘GSE172364’ under the secure token ‘ktapiyewftqnbsb’ at: https://www.ncbi.nlm.nih.gov/geo/query/acc.cgi?&acc=GSE172364NCBI.
