# Supplementary material for: RNautophagic regulation of DNMT3a‐dependent DNA methylation by Linc00942 enhances chemoresistance in gastric cancer
Source: Clin Transl Med. 2023 Jul 21;13(7):e1337. doi: 10.1002/ctm2.1337 (PMC10359971; doi:10.1002/ctm2.1337)
Supplement: Supplementary file 3 — Supporting information [file CTM2-13-e1337-s002.docx]

**
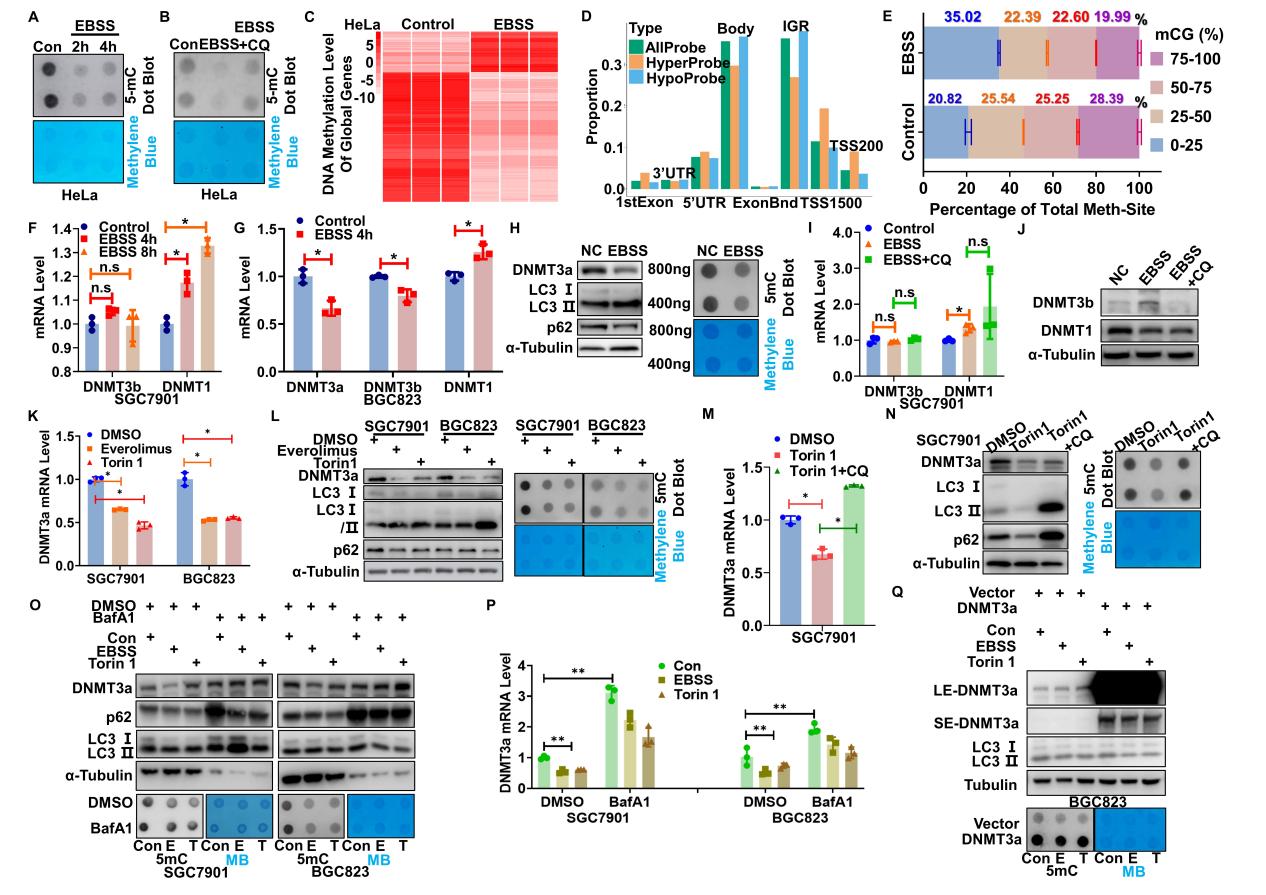
**

**Figure S1: Autophagy inhibits DNMT3a expression to remodel DNA methylation.**

**(A)-(B):** The effect of autophagy induction using EBSS treatment, together with or without CQ (autophagy inhibitor), total DNA 5-mC level in HeLa cells which was identified by dot blot assay with the methylene blue staining as the DNA loading reference. **(C):** The heat map of global DNA methylation in HeLa cells before and after autophagy activation by EBSS, which was detected using MethylationEPIC BeadChip. **(D):** Average DNA methylation proportion of different genomic contexts (1stExon, 3’UTR, 5’UTR, Body, ExonBnd, IGR, TSS1500 and TSS200) was determined using All-, Hyper-, or Hypo-probe, respectively. **(E):** A global view of DNA methylation in HeLa cells before and after autophagy activation. A cumulative bar plot showed the proportions at four methylation levels (0-25%, 25-50%, 50-75% and 75-100%). mCG mean CG methylation. **(F)-(J):** The effect of autophagy induction using EBSS treatment, together with or without CQ (autophagy inhibitor), on mRNA, protein or DNA methylation levels of different DNA methyltransferase in SGC7901 and BGC823 cells were determined by qRT-PCR, WB or Dot Blot. Data were presented as the mean ± SD, n = 3. *p< 0.01 (Student’s t test). **(K)-(P):** The effect of autophagy induction using EBSS, Everolimus or Torin 1 (mTOR inhibitors) treatment, together with or without CQ and BafA1 (autophagy inhibitor), respectively, on DNMT3a and autophagy-related markers level in SGC7901/BGC823 were determined by WB. mRNA level was performed by qRT-PCR. The total DNA 5-mC level was estimated by dot blot assay with the methylene blue staining as the DNA loading reference. **(Q)** The effect of autophagy induction using Torin 1 or EBSS treatment, together with or without DNMT3a overexpression, on global DNA methylation level in BGC823 were estimated by 5mC dot blot assay as in L, N and O.


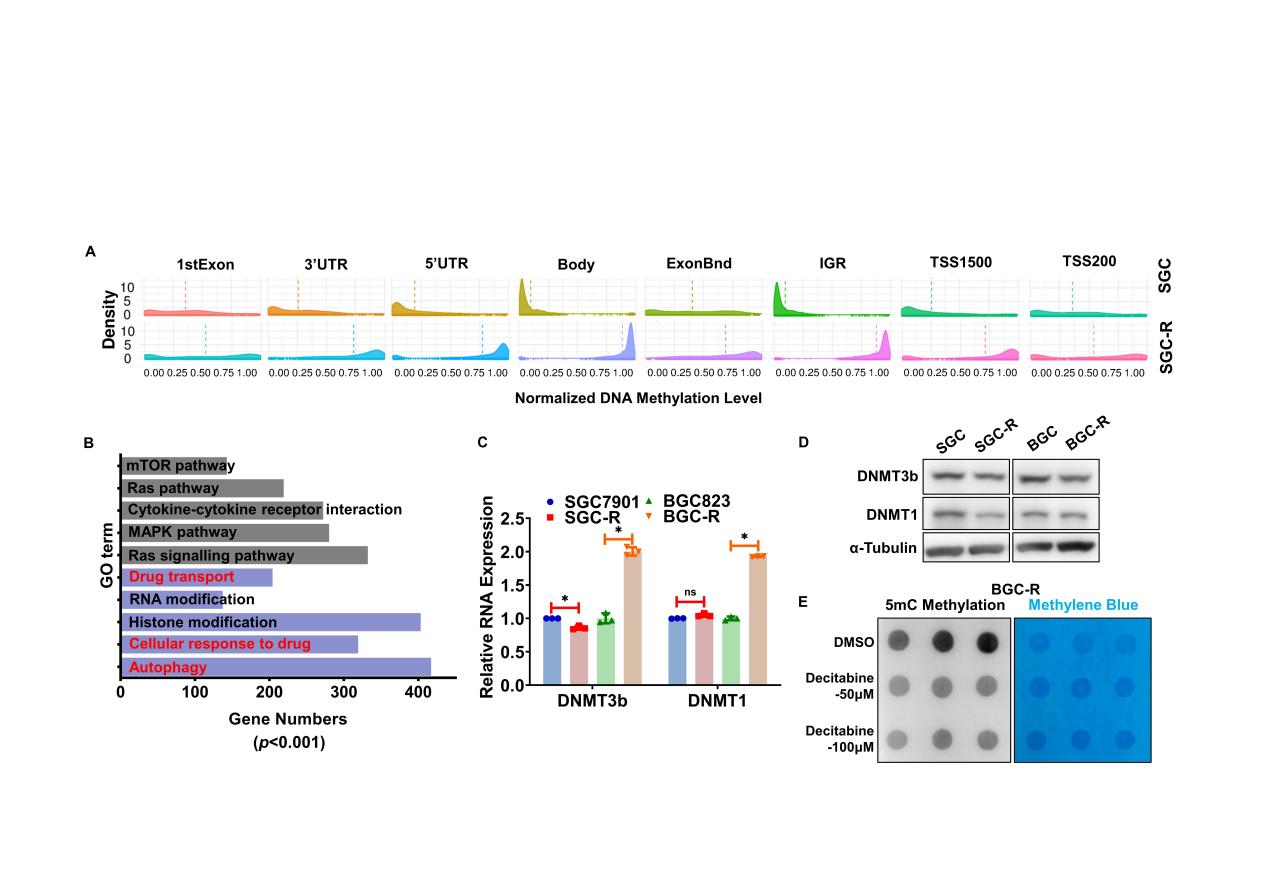


**Figure S2: Both DNMT3a expression and global DNA methylation are elevated in chemoresistant cancer cells.**

**(A):** Normalized DNA methylation density and level of different genomic contexts (1stExon, 3’UTR, 5’UTR, Body, ExonBnd, IGR, TSS1500 and TSS200) in SGC7901 and SGC-R cells.

**(B):** gene ontology (GO)results of most highly enriched (p<0.001) genes differentially expressed in SGC7901 and SGC-R cells.

**(C)&(D):** The expression of DNMT3b or DNMT1 in chemosensitive or resistant cells were detected by (C) qRT-PCR and (D) WB. Data were presented as the mean ± SD, n = 3. **p< 0.01 (Student’s t test).

**(E):** Dot blot of DNA isolated from DMSO and Decitabine (reduce levels of DNA methylation) treatment BGC-R cells.


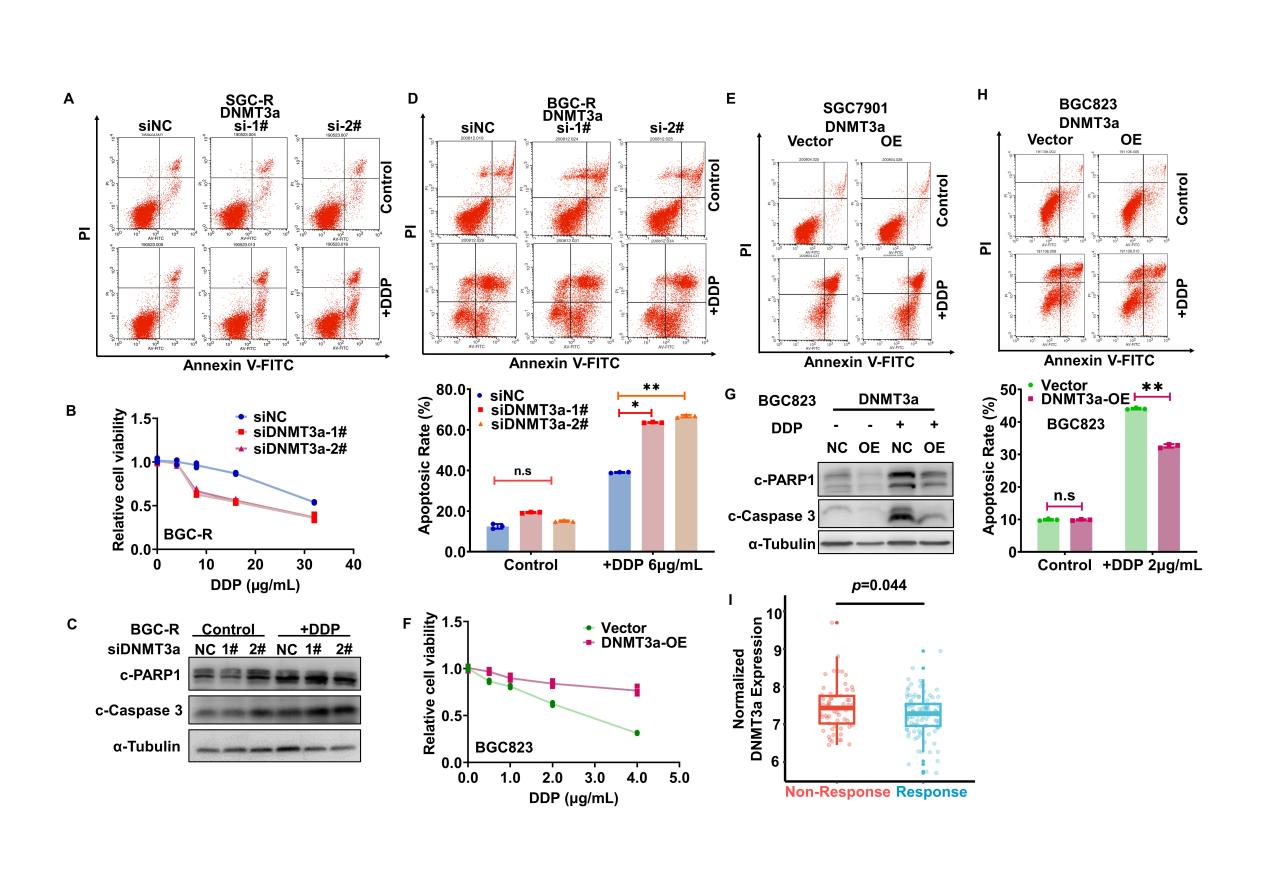


**Figure S3: Increased DNMT3a expression contributed to chemoresistance.**

**(A)&(E):** Raw data of flow cytometry assay as for DNMT3a knockdown in SGC-R and SGC7901 cells treated with or without DDP treatment for 24h.

**(B)-(D):** Effect of DNMT3a knockdown in BGC-R on the viability of resistant cells with or without DDP treatment for 24h were detected using MTS assay **(B)**, and apoptosis were analyzed using WB **(C)** as well as flow cytometry **(D)**.

**(F)-(H):** Effect of DNMT3a overexpression on the viability of sensitive cells with or without DDP treatment for 24h were detected using MTS assay (F), and apoptosis was measured using WB **(G)** as well as flow cytometry **(H)**.

**(I):** Exploring the association between DNMT3A expression and chemoresistance in gastric cancer patients form GSE62254 cohort. Chemotherapy-resistant (Non-Response) group was compared to chemotherapy-sensitive (Response) group (Wilcoxon test).


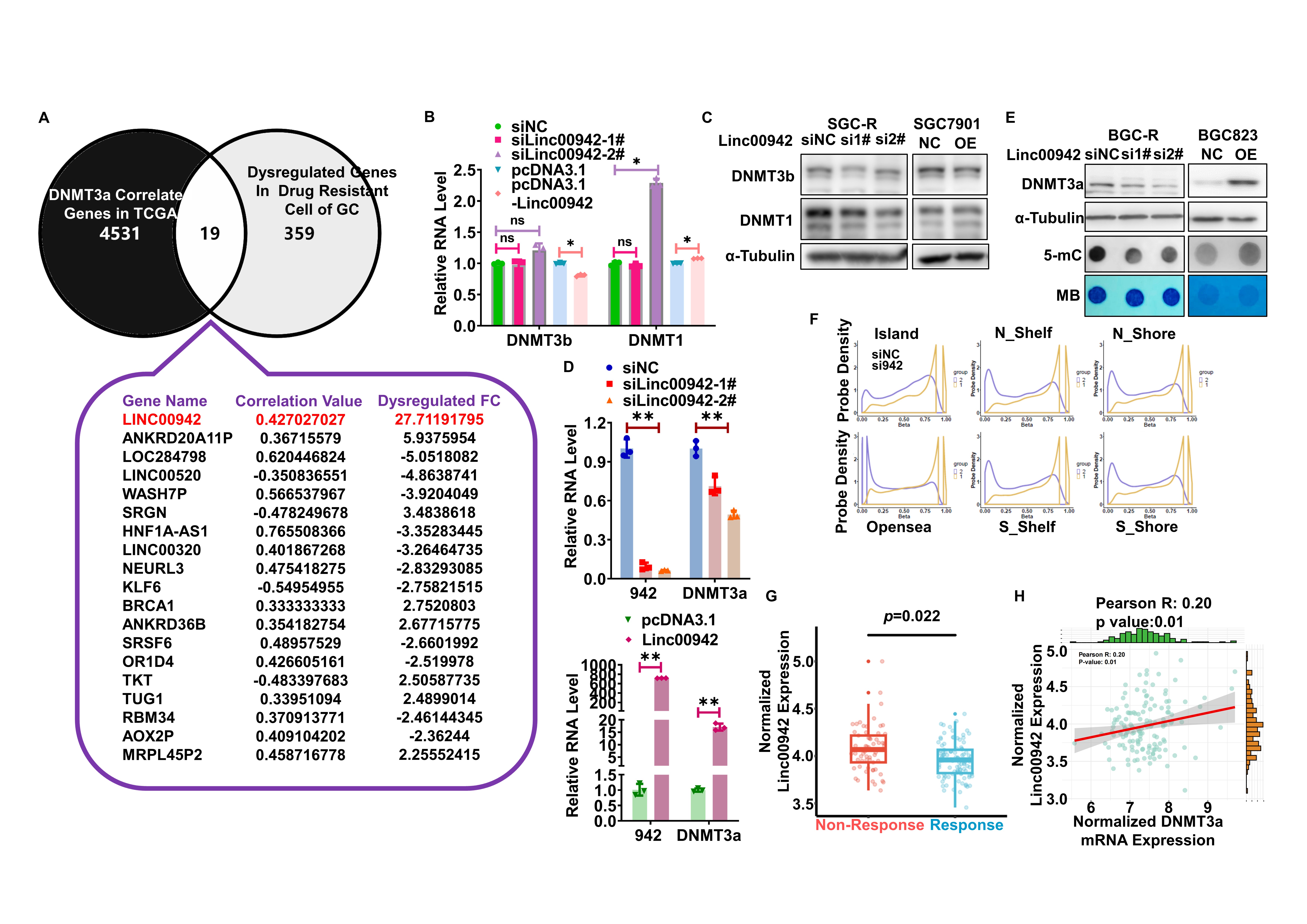


**Figure S4: Linc00942 increased the 5mC level through upregulating DNMT3a expression.**

**(A):** The detailed candidate genes correlated with DNMT3a expression and dysregulated in chemoresistant cells.

**(B)&(C):** Expression of DNMT3b/1 in SGC-R or SGC7901 cells transfected with Linc00942 siRNAs or overexpression plasmids were analyzed by qRT-PCR **(B)** and WB **(C)**. Experiments were all repeated three times and the representative data were shown. The asterisks indicate the statistical significance (p < 0.05)

**(D)&(E):** Expression of DNMT3a in BGC-R or BGC823 cells transfected with Linc00942 siRNAs or overexpression plasmids were analyzed by qRT-PCR **(D)** and WB **(E)**. Dot blot assay was performed to testify global DNA methylation level.

**(F)** The normalized DNA methylation density in different genomic contexts before (siNC) and after (si942) Linc00942 knockdown.

**(G):** Exploring the association between Linc00942 level and chemoresistance in gastric cancer patients form GSE62254 cohort. Chemotherapy-resistant (Non-Response) group was compared to chemotherapy-sensitive (Response) group (Wilcoxon test).

**(H):** The correlated relationship of Linc00942 and DNMT3a mRNA was analyzed using clinical data from GEO (GSE62254 cohort). Correlation coefficients were calculated by the Pearson and distance correlation analyses.


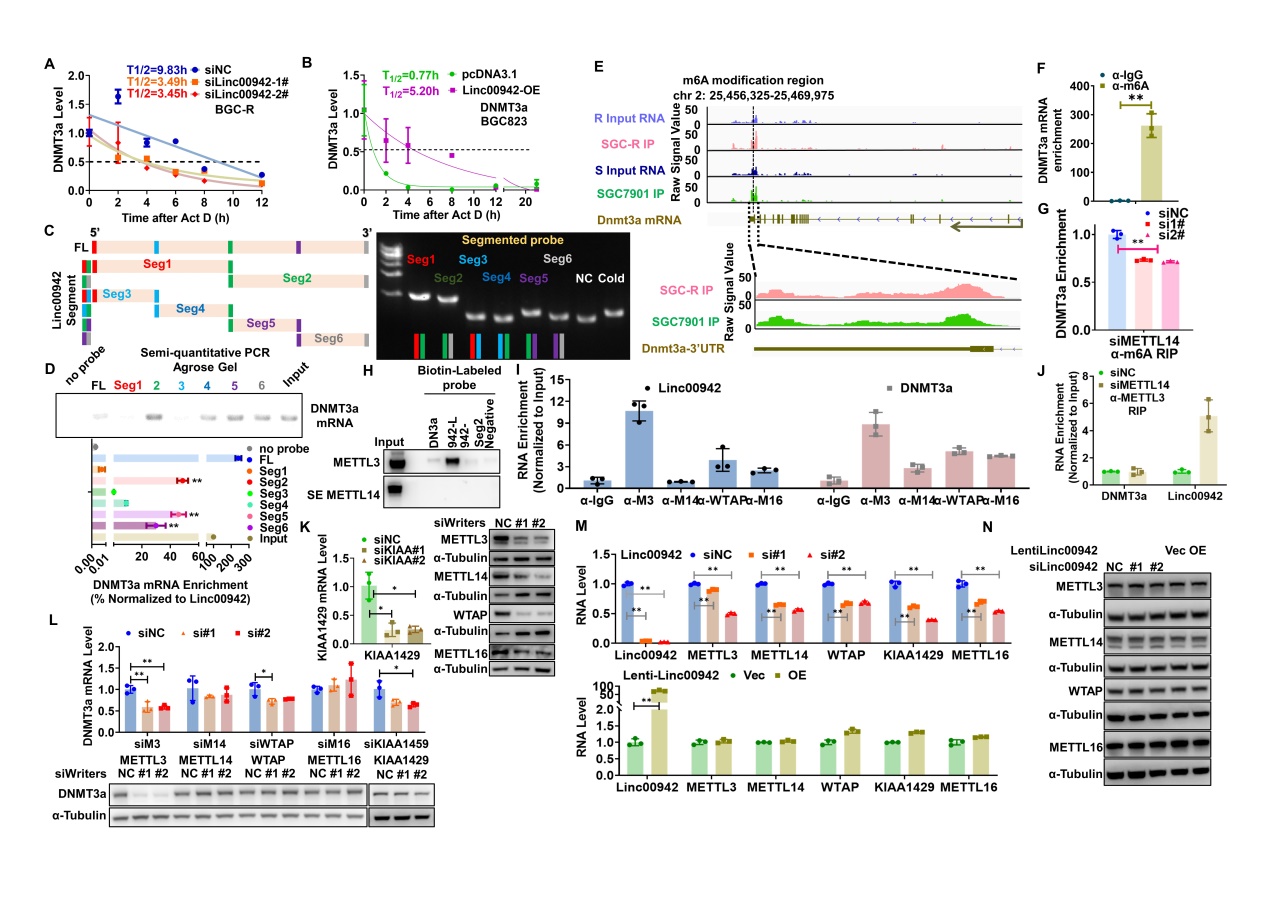


**Figure S5: Linc00942 stabilizes DNMT3a mRNA by recruiting METTL3 to facilitate its m6A modification.**

**(A)&(B):** DNMT3a mRNA half-life in BGC-Rs before and after knocking down Linc00942 levels **(A**) or in BGC823 cells with or without Linc00942 overexpression **(B**) were determined. Total RNAs from cells that were transfected with indicated siRNAs or plasmids were collected after Act D treatment for 0h, 2h, 4h, 6h, 8h and 12h, respectively.

**(C):** The gradient truncation segments of Linc00942 were synthesized by in vitro transcription and labeled with biotin. Agarose gel was used to identify the accurate position. **(D):** Biotin pull-down assay was operated to analyze the detailed binding region in Linc00942 for the interaction with DNMT3a mRNA. Agarose gel was performed using the samples after qRT-PCR. **(E):** Visualization of MeRIP-seq to show the m6A modification region in DNMT3a mRNA. **(F):** The enrichment observed for m6A immunoprecipitation was left that observed for IgG immunoprecipitation, indicating that the m6A antibody pulled down more DNMT3a mRNA than the non-specific IgG antibody.

**(G):** The effect of METTL14 knockdown on m6A modification enrichment changes of DNMT3a was assessed by RIP assay. **(H)-(L):** The capacity of Linc00942 and DNMT3a mRNA as to bind writers, as well as the effect of knocking down writers expression on Linc00942 and DNMT3a level was assessed by RNA pull down **(H),** RIP **(I),** qRT-PCR (K&L) and WB **(K&L). (J):** RIP assay was performed to identify the capacity of METTL3-DNMT3a/Linc00942 interaction or RNA stability changes upon knocking down METTL14. Normalized to Input, respectively. **(M)-(N):** The effect of Linc00942 knockdown or overexpression on changes of methyltransferase level was assessed by qRT-PCR&WB. Experiments were all repeated three times and the representative data were shown. The asterisks indicate the statistical significance (p < 0.05)


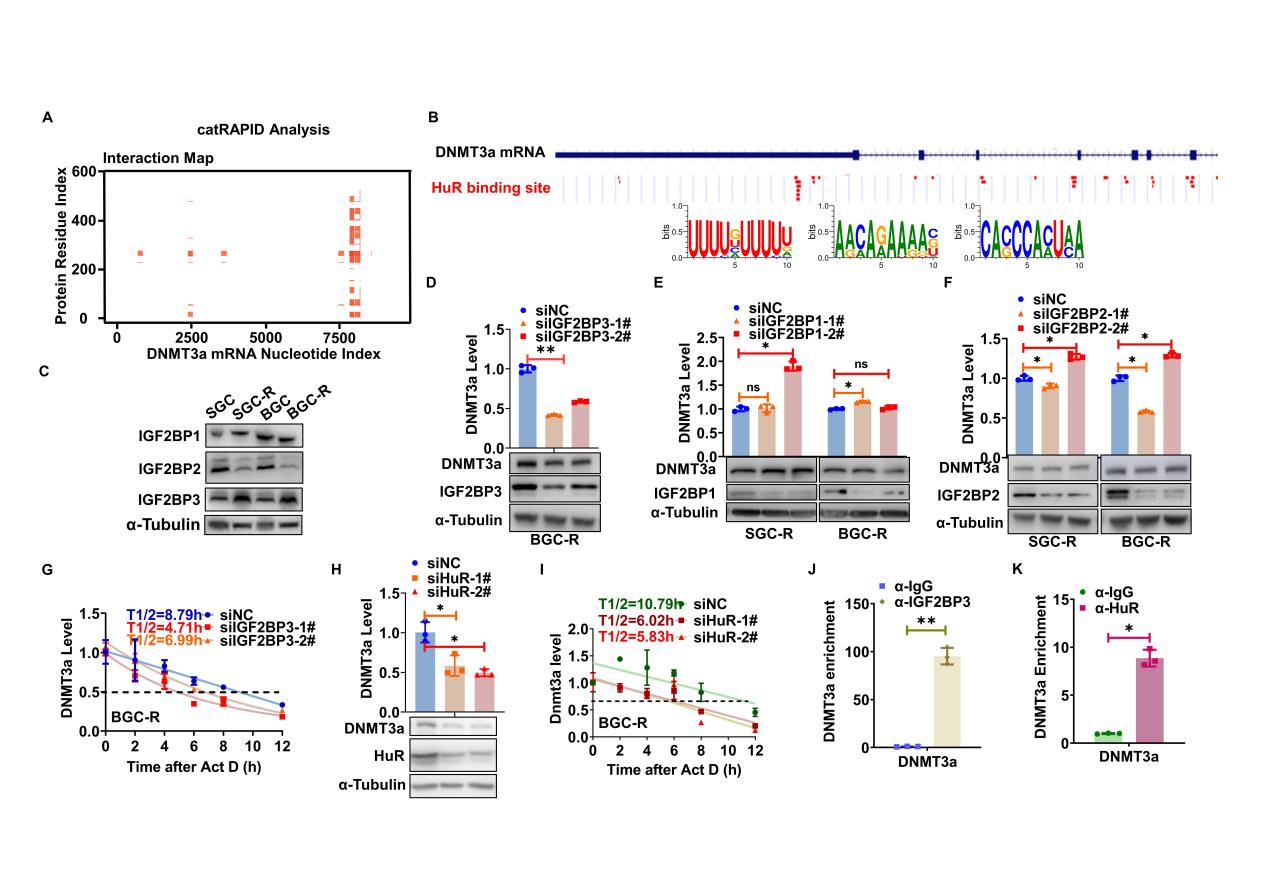


**Figure S6: Linc00942 enhanced IGF2BP3/HuR dependent stabilization of DNMT3a mRNA.**

**(A):** The detailed interaction Map between DNMT3a mRNA and IGF2BP3 according to the RNAInter database.

**(B):** The predicted binding site of DNMT3a mRNA to HuR in UCSC browser.

**(C):** The expression of IGF2BP family members in chemosensitive or resistant SGC7901 and BGC823 cells was detected by WB.

**(D)-(F):** Effect on expression changes of DNMT3a mRNA and protein upon knocking down IGF2BP family proteins or level in SGC-R and BGC-R cells were detected by qRT-PCR and WB, respectively.

**(G):** The effect of IGF2BP3 KD on DNMT3a mRNA stability in BGC-R cells.

**(H):** Effect on expression changes of DNMT3a mRNA and protein upon knocking down HuR were detected by qRT-PCR and WB, respectively.

**(J)&(K):** The interaction of IGF2BP3 or HuR with DNMT3a mRNA was analyzed by RNA-immunoprecipitation.


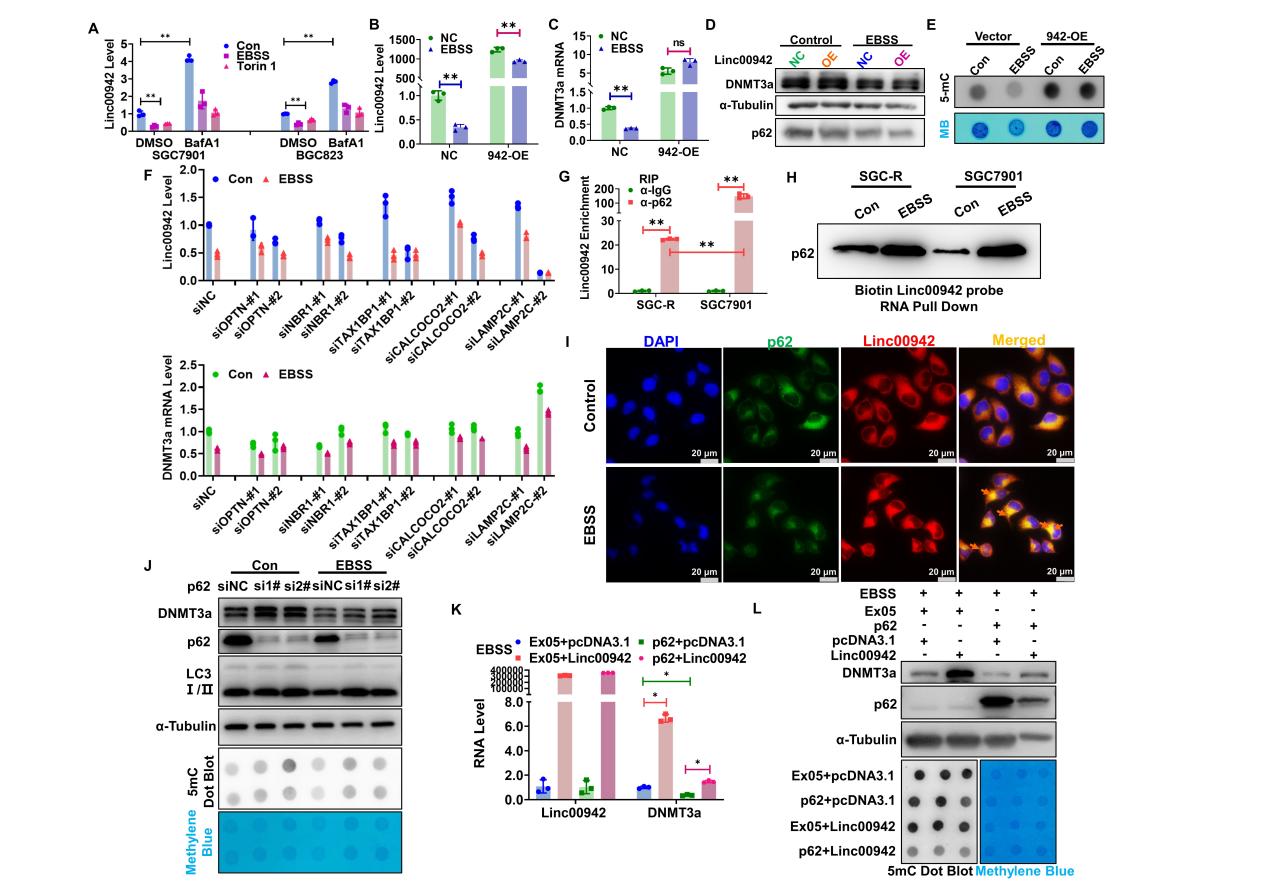


**Figure S7: p62 mediated RNautophagic degradation of Linc00942.**

**(A):** The effect of autophagy induction using EBSS or Torin 1 treatment, together with or without BafA1 (autophagy inhibitor), on Linc00942 level in SGC7901/BGC823 were determined by qRT-PCR.

**(B)-(E):** Effect of Linc00942 on DNMT3a mRNA, protein Expression and global DNA methylation level before and after EBSS treatment were determined by qRT-PCR, WB and 5mC Dot Blot assay, respectively.

**(F):** Effect of knocking down adaptor proteins combined with inducing autophagy through EBSS treatment on dynamic changes of Linc00942 and DNMT3a mRNA level was detected by qRT-PCR.

**(G)****-(H):** The interaction of p62 with Linc00942 was determined by RIP **(G)** and RNA pull down **(H)** both in SGC7901 and SGC-R cells.

**(I):** The colocalization of Linc00942 with p62 in SGC-R cells before and after EBSS treatment were analyzed by combined FISH and IFC assay (original magnification, 100X). Scale bar: 20μm.

**(J):** Effect of p62 knockdown on DNMT3a Expression and global DNA methylation level before and after EBSS treatment were determined by WB and 5mC Dot Blot assay, respectively.

**(K)-(L)** Effect of Linc00942 on DNMT3a mRNA, protein Expression and global DNA methylation level with or without p62 overexpression were determined by qRT-PCR (I), WB and 5mC Dot Blot assay (J), respectively.


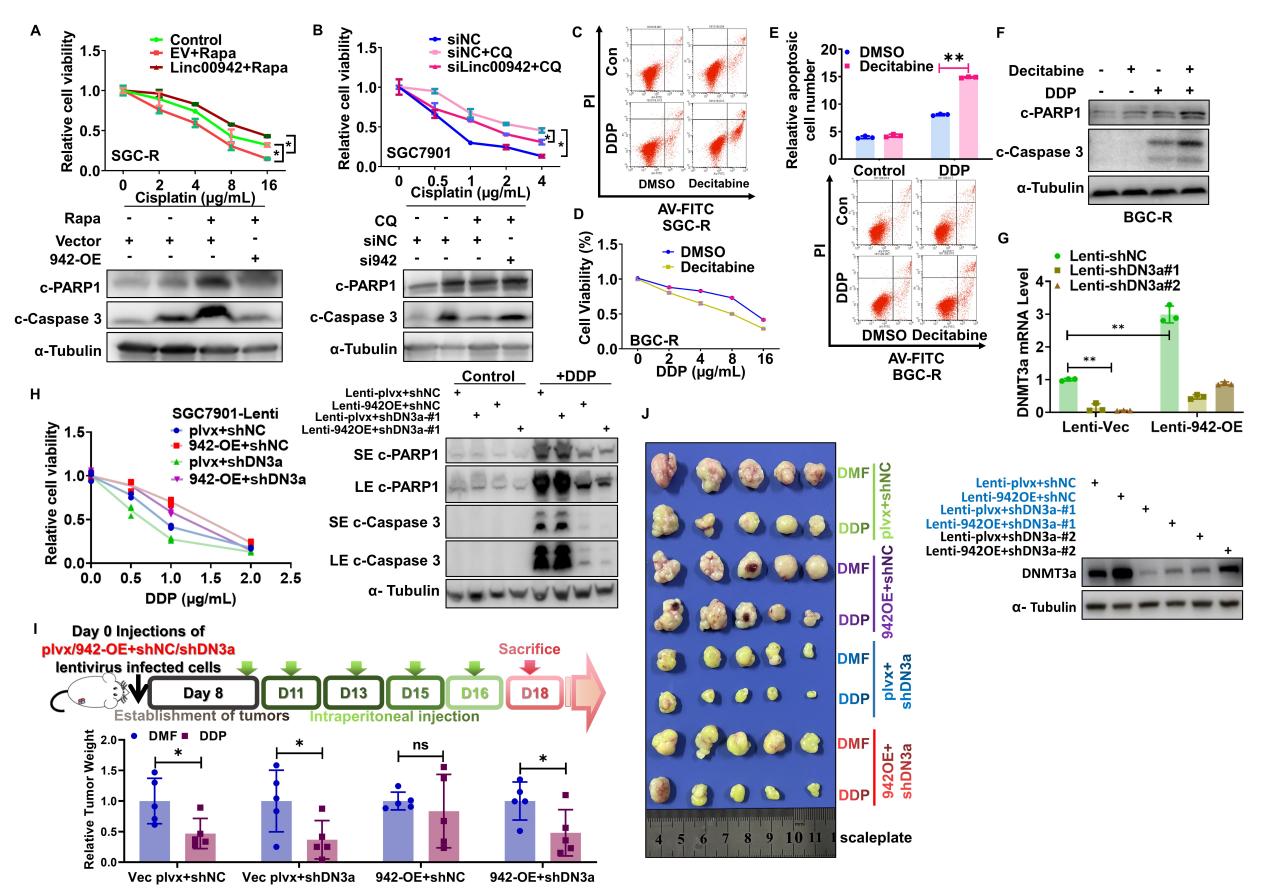


**Figure S8: Autophagic regulation of DNA methylation is relevant to chemoresistance.**

**(A)-(B):** The viability and apoptosis of SGC-R cells **(A)** or SGC7901 cells **(B)** treated as indicated were detected using MTS or WB, respectively. The asterisks indicated the statistical significance (p< 0.05).

**(C):** Raw data of Flow Cytometry to assess the apoptosis of SGC-R cells using DNMT3a inhibitor Decitabine before and after DDP treatment for 24h.

**(D)-(F):** Effect of DNMT3a inhibitor Decitabine on the viability or apoptosis of BGC-R cells with or without DDP treatment for 24h, was assessed by MTS assay **(D)**, Flow Cytometry **(E)** and WB **(F)**.

**(G):** The stable transfection cell line was constructed afresh and the efficiency of knocking down or overexpression was determined by qRT-PCR and WB.

**(H):** Effect of plvx+shNC, 942-OE+shNC, plvx+shDNMT3a and 942-OE+shDNMT3a on the viability or apoptosis with or without DDP (1μg/mL) treatment for 24h, was assessed by MTS assay **(Left)** and WB **(Right)**.

**(I)-(J):** The effect of DMF or DDP (2-4mg/kg) on tumor growth of SGC7901 cells with or without Linc00942 overexpression/ knocking down DNMT3a was determined by xenografts assay in nude mice (n=5/group). The injection cycle of DDP or DMF is shown in the illustration. Representative photographs of tumors excised from the mice, and the terminal weight on day 18 are shown in I (the relative terminal weight for tumor which was normalized to DMF in each group, respectively), J (photographs of tumors excised).
